# The MODNAP benchmark set for modeling ribose-based modified nucleic acid and nucleic acid–protein complexes: a case study with AlphaFold 3

**DOI:** 10.1093/nar/gkag999

**Published:** 2026-10-15

**Authors:** Jiemin Fu, Valeria Tohver Milam, Andrew C McShan

**Affiliations:** School of Chemistry and Biochemistry, Georgia Institute of Technology, Atlanta, GA 30332, United States; School of Materials Science and Engineering, Georgia Institute of Technology, Atlanta, GA 30332, United States; School of Chemistry and Biochemistry, Georgia Institute of Technology, Atlanta, GA 30332, United States

## Abstract

Modified nucleic acids play important roles in biology and biotechnology. However, their chemical and structural diversity as well as effects on nucleic acid structure and intermolecular interactions remain incompletely characterized. The performance of structure prediction and co-folding algorithms for modeling modified nucleic acids and nucleic acid–protein complexes has also not been evaluated. Here, we systematically analyze ribose-based modified nucleotides present in structures deposited in the Protein Data Bank. Modified nucleotides are primarily localized to the nucleobase, where they exhibit chemical and structural diversity shaped by molecular size, topological shape descriptors, and polarity-related properties. We develop MODNAP, the first curated dataset of experimentally determined structures containing modified nucleotides. Using MODNAP, we benchmark AlphaFold 3 on 1039 nucleic acid and nucleic acid–protein complex structures spanning a range of nucleotide modifications, tertiary folds, and biological functions. AlphaFold 3 achieves success rates exceeding 80% at an RMSD threshold of 2.5 Å. However, its accuracy in positioning modified nucleotides is variable and decreases for structures not explicitly represented in training. PoseBusters analysis reveals frequent intramolecular validity failures, primarily due to non-ideal ribose ring geometries. Together, these results establish MODNAP as a resource for evaluating and improving structure prediction methods for modified nucleic acids.

## Graphical Abstract

**Graphical Abstract:**
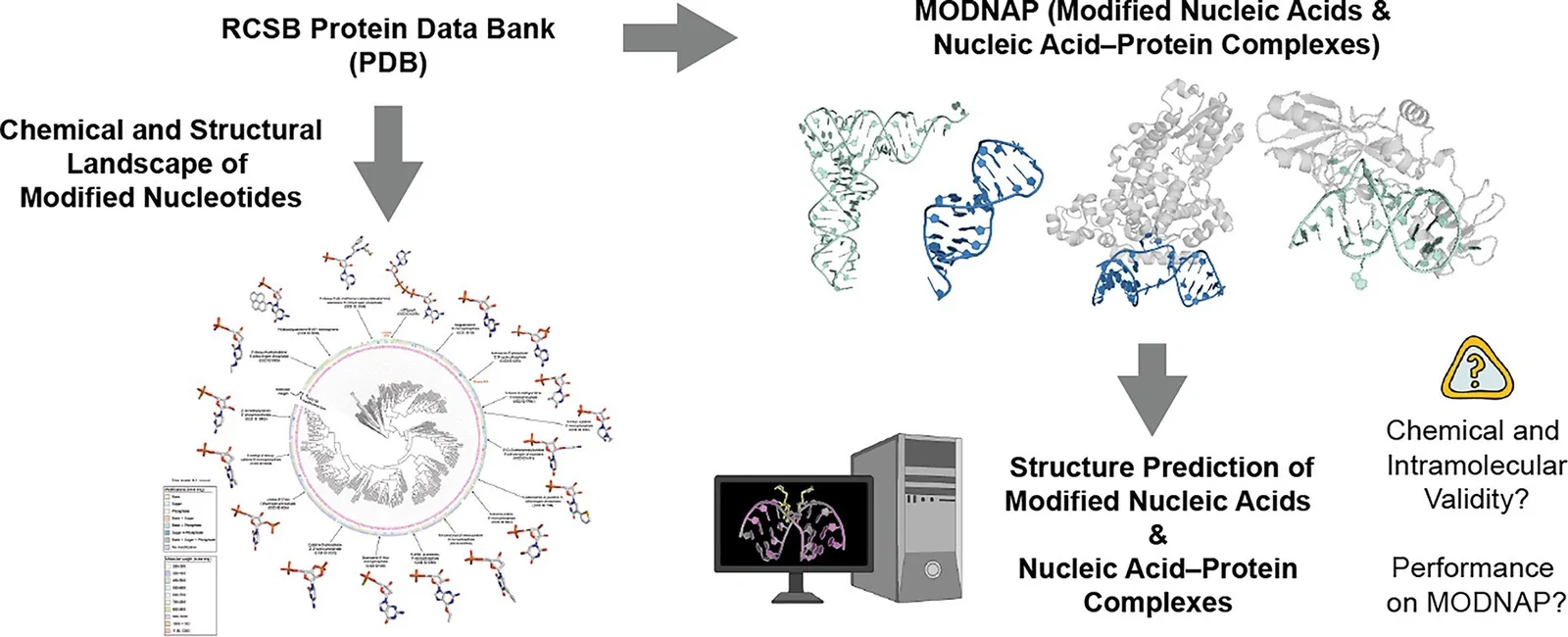

## Introduction

Modified nucleic acids contain analogs of standard nucleotides with alterations in the ribose sugar, the nucleobase, and/or the phosphate backbone [1, 2]. In cells, modified nucleic acids are typically generated enzymatically, although they can arise non-enzymatically from oxidative stress or chemical damage [3]. Online databases, such as DNAmod, RNAMDB, and MODOMICS, catalog >100 unique modified nucleotides present in endogenous RNA and DNA molecules [4–6]. Although relatively uncommon, modified nucleic acids play key roles in biology, including regulation of gene expression [7], epigenetics [8], translation [9, 10], RNA splicing [11], DNA repair [12], genome/RNA stability [13, 14], and cellular differentiation [15]. The most abundant DNA modification in mammals, 5-methylcytosine, accounts for ∼1% of the total DNA bases and is integral to transcriptional and epigenetic control [16–18]. The most abundant modification in mRNA, N6-methyladenosine, accounts for ∼0.4% of adenosine bases and contributes to mRNA stability, splicing, and translation [19, 20]. Other types of RNA, such as rRNA, tRNA, miRNA, and lncRNA, are also heavily modified to influence their stability, folding, and function [21–23]. Nucleic acid modifications also shape the landscape of biomolecular structure, stability, and conformational plasticity, which ultimately influences nucleic acid–nucleic acid, nucleic acid–protein, and nucleic acid–small molecule interactions [24–27]. Finally, modified nucleotides have defining roles in disease, including cancer [28, 29], immunity [30, 31], pathogen infection [32], and neurodegenerative disease [33].

Apart from naturally occurring nucleotide modifications, synthetic, non-natural nucleic acids, referred to as xeno nucleic acids (XNAs), have been developed for clinical applications, diagnostics, and biotechnology [34, 35]. XNAs feature modifications to the sugar, nucleobase, and/or phosphate backbone that confer enhanced chemical and thermal stability, binding specificity, or resistance to nucleases [36]. Examples of XNAs include (i) arabino nucleic acids, in which the ribose sugar is replaced with arabinose [37], (ii) Hachimoji nucleic acids, which incorporate synthetic nucleobases that expand the genetic code [38], and (iii) phosphorothioate nucleic acids, where a non-bridging oxygen in the phosphate backbone is replaced with sulfur [39]. These types of modifications expand the chemical, structural, and functional features of nucleic acids, enabling novel therapeutic and biotechnological applications [40, 41]. XNAs can also be used to generate XNAzymes, synthetic enzymes composed of modified nucleic acids that catalyze specialized biochemical reactions [42]. Despite these remarkable advances, the structural and functional impacts of natural and synthetic nucleic acid modifications remain incompletely understood, motivating the development of alternative approaches to predict their effects on DNA, RNA, and XNA structures, as well as their interactions with other biomolecules.

Numerous efforts have focused on computationally predicting the structures of unmodified nucleic acids, nucleic acid–protein, and nucleic acid–small molecule complexes [43–47]. However, very few approaches enable three-dimensional modeling of atomic structures of modified nucleic acids or nucleic acid complexes [48]. This bottleneck has recently begun to ease with the development of AlphaFold 3 and related AI-based methods, which enable accurate structure prediction for many types of biomolecular systems [49–52]. In contrast to two-dimensional models, such as UNAFOLD and NUPACK [53, 54], which rely on canonical base-pairing interactions, AlphaFold 3 predicts three-dimensional atomic structures of biomolecules and biomolecular complexes directly from user-provided sequences: amino acid sequence for proteins, nucleotide sequence for nucleic acids, and chemical component dictionary identification numbers (CCD ID) for modified residues. Although AlphaFold 3 excels at modeling canonical nucleic acids and their complexes [49, 55–59], its performance on systems containing modified nucleotides remains largely unevaluated. Abramson *et al*. reported success of AlphaFold 3 on a DNA structure containing 5-methylcytosine (PDB ID 7SDW, RMSD 0.50 Å, CCD ID 5CM) [49]. However, whether AlphaFold 3 can accurately model the broad diversity of nucleotide modifications across different nucleic acid folds remains unclear. We attribute this knowledge gap, in part, to the lack of curated datasets comprising high-resolution experimental structures of modified nucleic acids and nucleic acid complexes.

Here, we systematically evaluate the landscape of ribose-based modified nucleotides present in the Protein Data Bank (PDB) [60]. We identify a broad chemical space that reflects the extensive structural and functional diversity of nucleic acid modifications in both natural and synthetic biochemistry. We then develop MODNAP (Modified Nucleic Acids & Nucleic Acid–Protein complexes), the first curated dataset of experimentally determined atomic structures from the PDB containing modified nucleotides. MODNAP is designed for benchmarking and/or training structure prediction methods against nucleic acids containing modified nucleobases, sugars, and/or phosphates. We leverage MODNAP to perform a benchmark of AlphaFold 3 performance across 1039 nucleic acid and nucleic acid–protein complex structures spanning different nucleotide modifications, tertiary architectures, and biological functions. The analysis provides an assessment of the current capabilities and limitations of state-of-the-art structure prediction and co-folding methods for modeling modified nucleic acids. By establishing a standardized dataset, MODNAP provides a foundation for future method development, evaluation, and training efforts aimed at improving the prediction of nucleic acid structures containing nucleotide modifications.

## Materials and methods

### Construction of the MODNAP dataset

In-house Python scripts extracted CCD IDs from components.cif of PDB (https://www.wwpdb.org/data/ccd) using the keywords “deoxyuridine,” “deoxycytidine,” “deoxythymidine,” “deoxyguanosine,” “deoxyadenosine,” “adenosine,” “cytidine,” “guanosine,” “uridine,” and “thymidine.” For each CCD ID, information on the PubChem IDs and Simplified Molecular Input Line Entry System (SMILES) string [61] were obtained. Extraction on a date of September 18, 2025 resulted in a total of 1109 CCD IDs corresponding to both modified and unmodified nucleotides and nucleosides. Each of the 1109 CCD IDs corresponds to a unique nucleotide identity rather than a PDB entry. For example, CCD ID A23 (https://www.rcsb.org/ligand/A23) corresponds to adenosine-5′-phosphate-2′,3′-cyclic phosphate. Modification positions (sugar, nucleobase, and/or phosphate backbone) were mapped for all 1109 CCD IDs. Of these, 375 CCD IDs were integrated covalently into nucleic acid polymers (“is found in a polymer sequence”), and the rest were present as standalone ligands lacking covalent incorporation into nucleic acid polymers (“is found as a standalone ligand”). Here, the term “ligand” is used to refer to a nucleotide treated as a bound small-molecule ligand rather than as part of a nucleic acid polymer. All nucleotides are derived from either ribose, deoxyribose, or a modified version of these sugars (i.e. no other sugars, such as arabinose or galactose, are present in the dataset).

Atomic coordinates of legacy PDB (.pdb) files associated with each of the 375 CCD IDs of modified nucleotides incorporated nucleic acid polymers were fetched from the PDB with several filtering criteria [62]. First, nuclear magnetic resonance spectroscopy structures were not considered because they are calculated as conformational ensembles rather than a single coordinate model, introducing ambiguity and increased uncertainty into the atomic coordinates [63]. Second, X-ray or cryo-electron microscopy structures with an atomic resolution of 3.5 Å or less were considered. A resolution threshold of 3.5 Å or better was chosen because it provides sufficient accuracy to reliably model the placement of nucleotide functional groups and their interactions with surrounding molecules [64]. Third, molecules were only considered if the nucleic acid sequence length was ≤450 nucleotides and/or protein sequence length was ≤2400 amino acids to stay within AlphaFold 3’s practical inference limits, where prediction accuracy and convergence can deteriorate for larger systems [57]. Fourth, structures containing ambiguous residues (labeled “X” in the sequence information) or unmodeled sequence gaps were discarded. The full MODNAP benchmarking set consists of 1039 PDB entries corresponding to 129 unique CCD IDs representing different modified nucleotides. Of the 1039 structures present in the full MODNAP benchmark set, 103 were deposited to the PDB after September 30, 2021, the cutoff date used by AlphaFold 3 training and calibration [49]. These 103 structures are referred to as the MODNAP test set (post-cutoff date). In-house Python scripts analyzed the composition of the MODNAP benchmark set. Open access to MODNAP is provided on GitHub in a link provided in the “Data availability” statement.

### Systematic analysis of the chemical landscape of modified nucleotides in the PDB

For 2D stereochemistry-ambiguous clustering, SMILES strings and PubChem IDs for nucleotide CCD IDs were uploaded to ChemMine Tools [65]. Physicochemical properties were obtained in ChemMine Tools with the JOELib descriptor application, which computes a total of 38 descriptors (https://sourceforge.net/projects/joelib/). Using all JOELib descriptors, the nucleotides were clustered based on structural and physicochemical similarities via hierarchical clustering with the single linkage method, yielding a Newick tree file of the resulting clusters. The Newick tree was then imported into Interactive Tree of Life (iTOL) v7.4.1 to generate unrooted circular dendrograms [66]. Chemical 2D structures and nomenclature for CCD IDs were obtained directly from the associated PDB page; for example, https://www.rcsb.org/ligand/A23.

For 3D stereochemistry-aware clustering, ideal SDF structures for all unique nucleotide CCD IDs were retrieved from the RCSB Protein Data Bank Chemical Component Dictionary. Structures were parsed and stereochemistry was assigned from 3D coordinates using RDKit [67]. For each structure, two complementary descriptor sets were computed: a 2D Morgan fingerprint with chirality, capturing exact connectivity and stereochemical topology, and a USRCAT descriptor, capturing 3D shape and pharmacophore features [68]. Pairwise chemical distances were calculated as a weighted combination of Tanimoto distance between Morgan fingerprints (weight = 0.50), USRCAT shape distance (weight = 0.35), and a heavy-atom count size penalty to prevent clustering of mono- and di-nucleotides (weight = 0.15). The pairwise distance matrix was used to construct a hierarchical clustering tree via the unweighted pair group method with arithmetic mean using Biopython, yielding a Newick tree file of the resulting clusters. The Newick tree was then imported into Interactive Tree of Life (iTOL) v7.4.1 to generate dendrograms [66]. Ideal 3D chemical structures for CCD IDs were obtained directly from the associated PDB page; for example, https://files.rcsb.org/ligands/download/A23_ideal.sdf.

### AlphaFold 3 modeling

AlphaFold 3 models were generated using a locally installed version (v3.0.0, commit e2afc41) obtained from the publicly available repository (https://github.com/google-deepmind/alphafold3). AlphaFold 3 model parameters were provided by Google DeepMind for non-commercial use and downloaded on November 13, 2024. For each nucleic acid or nucleic acid–protein complex, a JSON input file was prepared containing the nucleic acid sequence, protein sequence, the modified nucleotide (CCD identifier), and in some cases ions and/or a small molecule ligand (CCD identifier). Structure prediction inference was conducted using the run_alphafold.py script from AlphaFold 3 under default parameter settings. Briefly, the diffusion-based generative module was executed with 200 denoising steps per sampling trajectory. Iterative refinement of model representations was performed with a recycling depth of 10 (num_recycles = 10). Predictions were computed using a single random seed (modelSeeds = [10]). For each input, five independent diffusion trajectories were generated (num_diffusion_samples = 5). The resulting five structural models generated per sample were subsequently ranked according to the model predicted template modeling (pTM) or interface predicted template modeling (ipTM) scores. The top scoring model (high pTM or ipTM score) was selected for further analysis. All predictions were executed on compute nodes equipped with NVIDIA A100 GPUs, eight CPU cores, and 80 GB RAM. Example scripts to run AlphaFold 3 locally on a SLURM high-performance cluster are provided on GitHub in a link provided in the “Data availability” statement.

### Evaluation of AlphaFold 3 models against experimental structures

Atomic coordinates of AlphaFold 3 models were compared with those of experimental ground truth structures from the PDB using root mean square deviation (RMSD) calculated in PyMOL v3.1.6.1 following least-squares global alignment of the entire complex. RMSD quantifies atomic-level differences between superimposed predicted and experimental structures and reflects a global rigid-body alignment. RMSD values below 1.5 Å represent the highest-quality predictions, values from 1.5 to 2.5 Å indicate high-quality predictions, values between 2.5 and 4 Å correspond to intermediate-quality predictions, and values above 4 Å are considered failed predictions [69–71]. Using these standardized RMSD threshold values, the success rates among different types of molecules containing modified nucleotides were compared. Success rates were defined by the percentages of predicted nucleic acid and nucleic acid–protein complex poses that matched ground truth structures below specified RMSD threshold values (2, 2.5, 3 Å). Template modeling score (TM-score) and local distance difference test (lDDT) structure comparison values were calculated in OpenStructure v2.11.0 [72]. TM-score measures structural similarity using distances between corresponding atoms scaled by a length-dependent normalization factor and reflects a global structural superposition, where values above 0.6 generally indicate likely accurate predictions [73, 74]. lDDT measures the fraction of preserved local all-atom distances without requiring structural superposition and evaluates local structural accuracy within defined distance tolerance thresholds, where values above 0.6 generally indicate likely accurate predictions [75]. Interaction network fidelity (INF) values INF_all and INF_WC structure comparison values were calculated as the geometric mean of precision and sensitivity (INF = $\sqrt {PPV \times \textit{Sensitivity}} $), by comparing predicted and experimental nucleic acid interaction networks [76]. Precision (PPV) was defined as the fraction of correctly predicted interactions among all predicted interactions, PPV = TP/(TP+FP), where TP and FP denote true positive and false positive base-pair interactions, respectively. Sensitivity (recall) was defined as the fraction of experimentally observed interactions that were correctly recovered by the prediction, Sensitivity = TP/(TP+FN)​, where FN denotes false negative interactions. INF_WC considers only Watson–Crick base pairs, whereas INF_all includes both canonical and non-canonical base-pairing interactions. INF metrics (INF_all and INF_WC) measure the accuracy of predicted nucleobase interactions relative to the experimental structure, where INF_all considers all interaction types and INF_WC considers only Watson–Crick base pairs specifically; values range from 0 to 1 with higher values indicating greater fidelity to the experimental interaction network [76]. Interaction INF (I-INF) was calculated analogously to INF_all and INF_WC, but restricted to intermolecular contacts between two specified components of a complex (i.e. DNA–protein), rather than intramolecular base pairing as described by Ludwiczak *et al*. [77]. I-INF = √(PPV × Sensitivity) with TP, FP, and FN defined as interface contacts (hydrogen-bonded residue pairs) correctly predicted, predicted but not observed, and observed but not predicted, respectively.

### Structure validity checks with PoseBusters test suite

The PoseBusters test suite v0.6.5 was installed following the instructions from the GitHub repository (https://github.com/maabuu/posebusters) [78]. For PoseBusters analysis, the modified nucleotide(s) corresponding to the target CCD IDs or natural unmodified nucleotides (A, C, T, G, U) were extracted from each AlphaFold 3-predicted structure using PyMOL and saved as an individual PDB file. The extracted nucleotide structures were then evaluated using the PoseBusters test suite. PoseBusters was used to assess molecular quality across two categories: (i) chemical validity, defined by successful molecule loading, sanitization, InChI convertibility, and absence of radical species; and (ii) intramolecular validity, defined by satisfaction of bond length and angle criteria, absence of internal steric clashes, correct aromatic and non-aromatic ring geometry, proper double-bond planarity, and internal energy consistency. An AlphaFold 3-generated model was considered valid within a category only if all constituent tests within that category were passed. Models passing all evaluated categories were classified as PB-valid (PoseBusters test suite valid).

### Structure entanglement checks with RNAspider

AlphaFold 3 predictions stored in mmCIF (.cif) format were filtered against the MODNAP entry metadata list to isolate structures containing RNA components (including RNA single strands, RNA duplexes, DNA–RNA hybrids, and RNA–protein complexes). Selected mmCIF files were converted to PDB format using GEMMI v0.6.7. Secondary structures in dot-bracket format (.dot) were generated via automated HTTP POST requests to the RNApdbee v3.0 REST API [79]. Analysis parameters submitted to the API utilized the RNAPOLIS engine, model selection set to the first model, pseudoknot handling enabled, and non-canonical base pair annotations. Topological entanglements were identified and categorized using RNAspider (spider_v72) [80, 81]. Paired .dot secondary structure and .pdb tertiary structure files were submitted sequentially to determine raw structural entanglements. Topological entanglements were identified and classified using RNAspider running under default parameters in mode 1. Structure tracing was mapped directly to P and C4′ backbone atoms with isolated base pairs removed. Structural analysis evaluated both intramolecular and intermolecular interactions, focusing on primary and secondary entanglement orders across structural element lengths ranging from 4 to 30 residues. Geometric interaction cutoffs were set to standard default thresholds, including a maximum hydrogen bond distance of 6.50 Å and a minimum spatial distance of 7.0 Å. The resulting output was parsed through RNAspider’s classification tool to generate an initial topological profile, from which multi-order entanglements were filtered out to isolate single-order structural entanglements.

## Results

### A systematic analysis of the landscape of modified nucleotides in the protein data bank

The chemical and structural landscape of modified nucleotides in the PDB, encompassing both natural and synthetic nucleotides, has not yet been systematically evaluated. To address this gap, a comprehensive list of all nucleotides with ribose, deoxyribose, or modified versions of these sugars present in the PDB was compiled. A total of 1109 nucleotides, of which 1059 were modified and 50 were unmodified, were identified. A subset of modified nucleotides (375/1059) were covalently incorporated into DNA or RNA polymers, whereas the remainder (754/1059) were present as standalone ligands not incorporated into nucleic acid backbones. Some modified nucleotides were found in both contexts, appearing both as polymer-incorporated residues and standalone ligands. Each of the 1109 nucleotides is represented by a unique CCD ID and SMILES string. To visualize the landscape of the 375 modified nucleotides covalently incorporated into nucleic acid polymers, we generated two complementary dendrograms: a 2D, stereochemistry-blind clustering based on JOELib physicochemical descriptors, and a chirality-aware clustering based on combined 2D/3D descriptors (Fig. 1 and Supplementary Fig. S1). Multiple classes of modifications were observed (indicated by the inner ring color): nucleobase, sugar, and phosphate modifications, as well as combinations thereof. Modification types represented in the dataset include dinucleotide dimers (CCD ID GTA), bases or sugars bearing bulky substituents (CCD IDs DG8, 31M, 75B), cyclic sugar modifications (CCD IDs CCC, A23), incorporation of non-canonical elements (Se, S, Br, and F; CCD IDs FMU, GS, 75B, 5BU), alkyl chain additions at the base or sugar (CCD IDs E6G, E3C, U31, D00, TTM), and bases with distinct redox properties (CCD ID IG) (Fig. 1 and Supplementary Fig. S1). The molecular weight of modified nucleotides (indicated by the outer ring color) ranged from 200 to 1200 Da with a mean molecular weight of 453 Da, higher than the canonical nucleotides A, G, C, T, and U, which range from 300 to 340 Da. Most modifications were located on the base, consistent with the activity of key modifying enzymes, such as methyltransferases, deaminases, demethylases, and glycosylases [82, 83].

**Figure 1.**
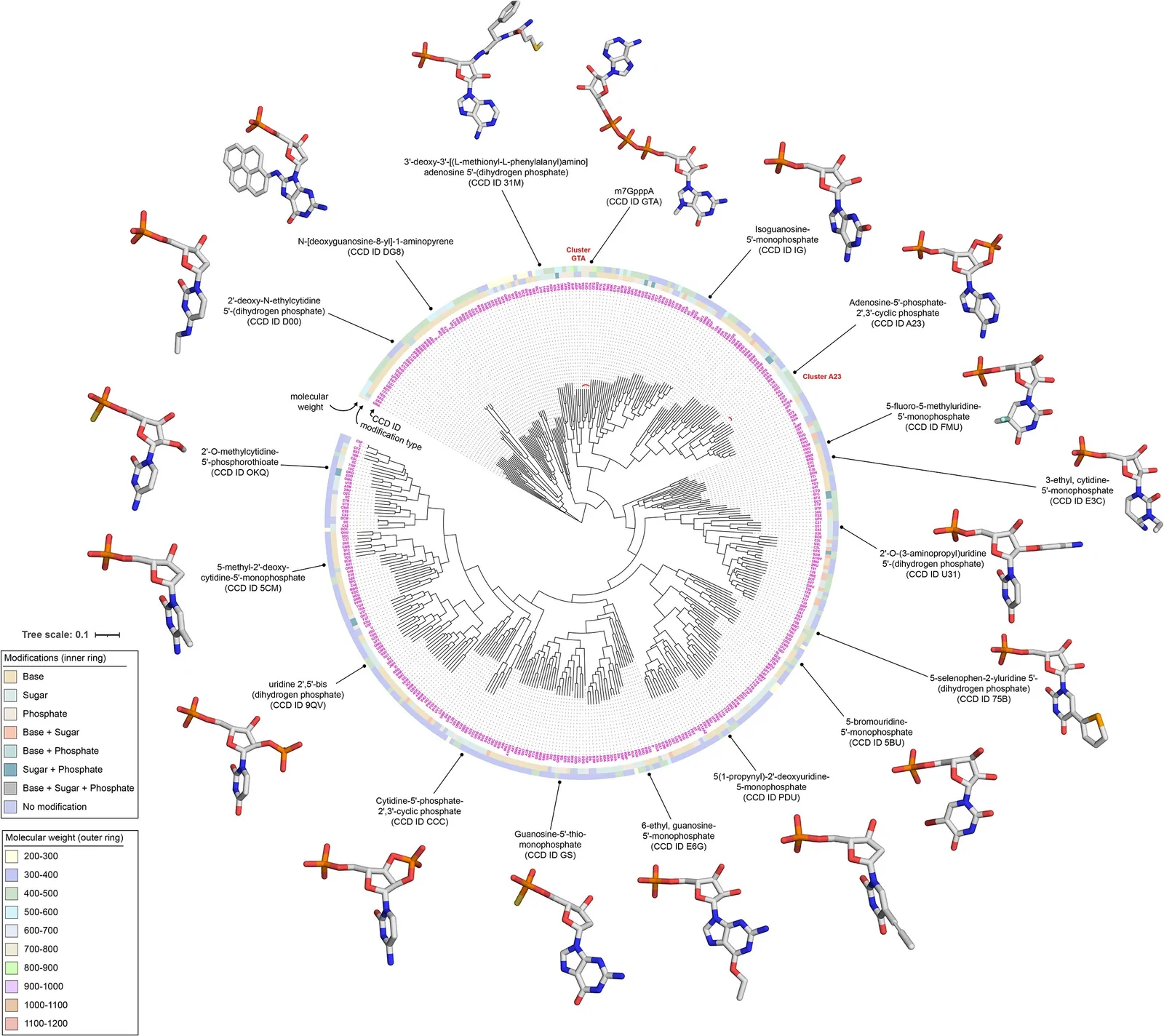
Landscape of modified nucleotides present in nucleic acid polymers in the Protein Data Bank. A circular dendrogram of 375 modified nucleotides incorporated within DNA or RNA polymers in the PDB. A few unmodified natural nucleotides (A, G, T, C, U) were included for reference. The dendrogram was generated in iTOL v7 from a Newick tree derived from unweighted pair group method with arithmetic mean clustering using chiral-aware 2D Morgan fingerprints and 3D USRCAT shape similarity of ideal nucleotide structures. Three-dimensional chemical structures of representative modified nucleotides were visualized in PyMOL with carbon, nitrogen, oxygen, and phosphorus atoms colored gray, blue, red, and orange, respectively. The dendrogram inner ring is colored by modification type, while the outer ring is colored by molecular weight (Daltons, Da). The two red arches by the cluster branches denote clusters described in the main text (GTA cluster, top; A23 cluster, top right).

Both dendrograms resolved distinct clusters with shared properties; two of which clusters are described below. In the 2D, stereochemistry-blind analysis, the physicochemical descriptors that most strongly guided clustering were molecular size-related features (number of bonds, number of atoms, molecular weight), topological features (Zagreb and Kier shape descriptors), and polarity-related properties (polar surface area, LogP) (Supplementary Fig. S2A–D). In the chirality-aware 2D/3D analysis, clustering was driven by bond connectivity, topological features, and stereochemical topology together with 3D shape similarity, producing a distinct tree topology (Fig. 1). Cluster A23 in the 2D tree includes CCD ID A23 (adenosine-5′-phosphate-2′,3′-cyclic phosphate) and AVC (adenosine-5′-monophosphate-2′,3′-vanadate) whose clustering is driven by similarities in physicochemical profiles (Supplementary Fig. S3). Cluster A23 in the chirality-aware tree also grouped with AVC, reflecting shared 3D/stereochemical features, such as ribose-cyclic phosphate geometry. The function of these modified nucleotides includes natural or synthetic 3′-terminal RNA cleavage products generated by ribozymes or ribonucleases [84]. A second cluster, cluster GTA, in both dendrograms includes CCD ID GTA (m7GpppA) and spans four CCD IDs originating from the same root. The molecules share a symmetric dinucleotide architecture connected via a 5′-5′ triphosphate bridge, with variations occurring at the nucleobase level, differing in either the identity of the 3′ nucleoside (adenosine versus guanosine) or the presence of an N7-methyl modification on the 5′ guanosine (Supplementary Fig. S4). m7GpppA and related modified nucleotides function as naturally occurring 5′ mRNA caps involved in transcript stabilization, trafficking, and translation initiation [85]. Overall, comparing the two dendrograms reveals that 2D and 3D/stereochemistry-aware analyses capture complementary aspects of the modified nucleotide landscape in the PDB, together spanning a broad and underappreciated structural and chemical space.

### Features and composition of MODNAP

The performance of AlphaFold 3 on modified nucleic acids and nucleic acid–protein complexes remains largely unexplored [49]. We attribute this, in part, to the lack of curated datasets of high-resolution atomic structures. Such a dataset could be used as ground truth to rigorously evaluate the robustness and accuracy of software for structure prediction, co-folding, and other modeling efforts. To address this bottleneck, we constructed MODNAP (Modified Nucleic Acids & Nucleic Acid–Protein complexes), a dataset of DNA, RNA, DNA–RNA hybrid, DNA–protein complex, and RNA–protein complex structures containing modified nucleotides. MODNAP contains a total of 1039 structures across 129 modified nucleotide CCD IDs that span a wide chemical and structural space. MODNAP entries containing DNA were primarily double-stranded, with a smaller fraction comprising single-stranded or multi-stranded DNA (Supplementary Fig. S5). Structures with RNA exhibited a more balanced distribution between single-stranded and double-stranded forms, with only a minor representation of multi-stranded RNA complexes. Many MODNAP entries contained at least one unmodified DNA or RNA strand. Among modified sequences, most DNA and RNA molecules carried a single unique CCD modification type, although a subset contained more than five distinct types. The majority of modified DNA and RNA sequences had fewer than 20% of their nucleotides modified; however, a notable proportion of RNA molecules consisted entirely of modified nucleotides (Supplementary Fig. S6). The dataset is skewed toward structures containing CCD IDs 5CM (5-methylcytidine, *n* = 172), 8OG (8-oxoguanosine, *n* = 90), and BRU (5-bromouridine, *n* = 64), which collectively account for ∼31% of all MODNAP entries (Supplementary Fig. S7). The majority of CCD IDs (88 of 129, ∼68%) are represented by five or fewer PDB structures, and 63 CCD IDs (∼49%) are represented by a single structure, reflecting an uneven structural coverage of the modified nucleotide landscape in the PDB. MODNAP includes 42.1% DNA–protein complexes, 24.8% RNA structures, 24.7% DNA structures, 6.7% RNA–protein complexes, and 1.7% DNA–RNA hybrids (Fig. 2A). MODNAP exhibits a non-uniform distribution of modified nucleobases with a bias toward cytosine (37.4%), uracil (24.3%), and guanine (23.3%), and comparatively lower representation of adenine (10.9%) and thymine (4.1%) (Fig. 2B). This pattern is consistent with the enrichment of both natural and synthetic modifications skewed toward C, U, and G in endogenous DNA and RNA molecules [86, 87]. Most modifications present in MODNAP entries were located on the base (66.9%) or sugar (27.9%) (Fig. 2C). This is consistent with observations that natural and synthetic nucleic acid modifications most commonly occur at base- or ribose-modifying positions accessible to enzymatic or chemical functionalization [88]. MODNAP contains nucleic acids ranging from 10 to 410 nucleotides in length and proteins ranging from 50 to 2350 amino acids, reflecting an enrichment for relatively small to moderately sized nucleic acid and protein components (Fig. 2D and E). Nucleic acid–protein complexes in MODNAP span a wide range of relative size, as reflected by the protein-to-nucleotide ratio (number of amino acids relative to number of nucleotides), with most structures containing proteins that are ~5- to 20-fold larger than their bound nucleic acids (Fig. 2F). Modified nucleic acids and nucleic acids–protein complexes span a broad range of functional classes, with 31% of entries corresponding to synthetic nucleic acids (oligonucleotides and aptamers) (Fig. 2G). Nucleic acid–protein complexes present in MODNAP are associated with nucleic acid metabolism (replication, transcription, and translation), nucleic acid modifying enzymes (hydrolases, transferases, lyases, and ligases), and gene expression regulation (nuclear hormone receptors, splicing factors, and quadruplex-binding proteins). MODNAP contains a representative distribution of common RNA, DNA, and nucleic acid–protein complex folds and tertiary structures, including A-DNA, B-DNA, Z-DNA, RNA helices, rRNA, tRNA, riboswitches, ribozymes, and G-quadruplexes (Supplementary Fig. S8).

**Figure 2.**
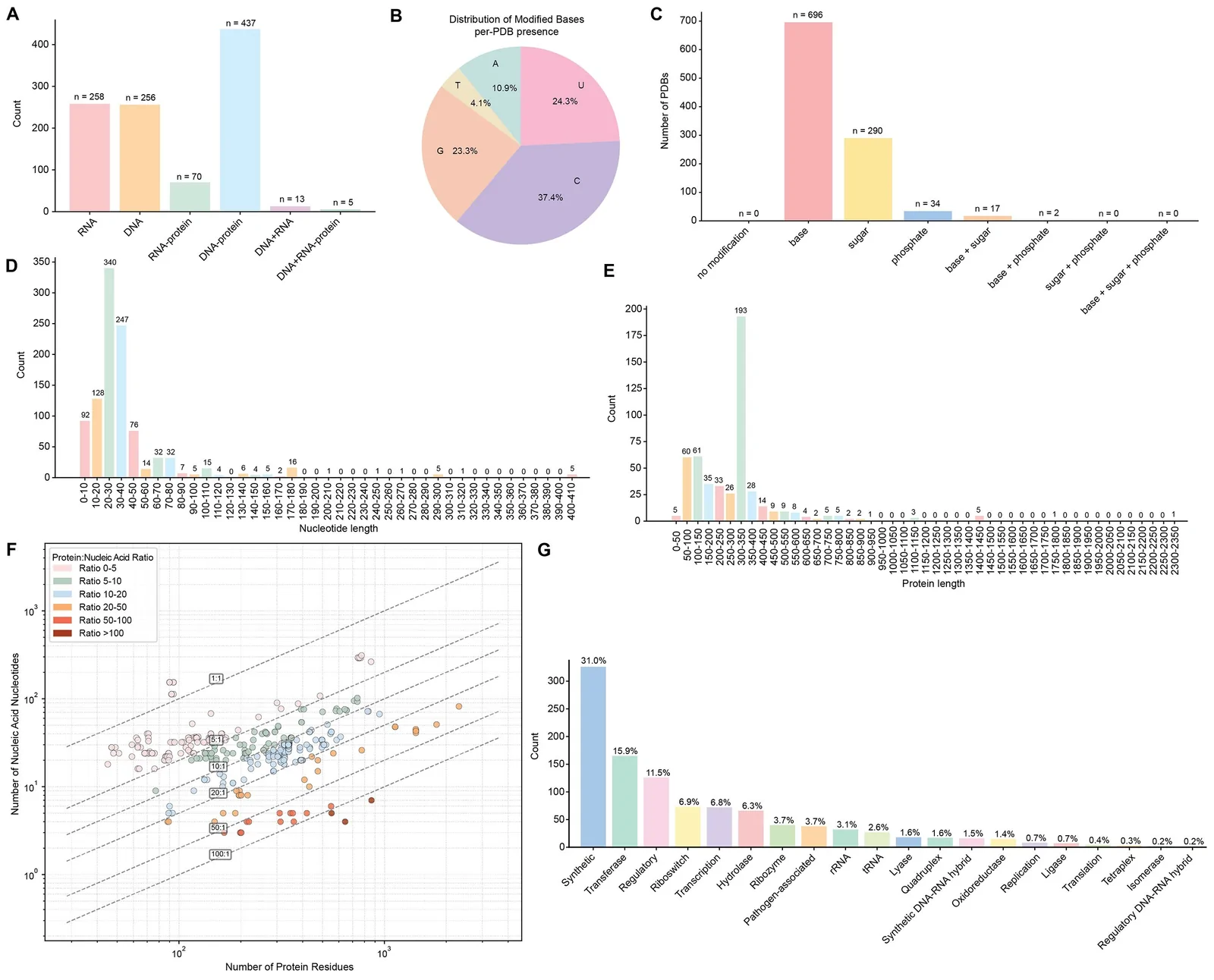
Features of the MODNAP benchmark set of modified nucleic acids and nucleic acid–protein complexes. (**A**) Total count of RNA, DNA, RNA–protein, DNA–protein, RNA–DNA hybrid, and DNA–RNA–protein structures in MODNAP. (**B**) Distribution of modified nucleobases (A, T, G, C, and U) by percentage in MODNAP. (**C**) Total number of PDB entries containing different modification types in MODNAP. (**D**) Total count of entries in MODNAP containing nucleic acid lengths in binned intervals of 10 nucleotides. (**E**) Total count of entries in MODNAP containing protein lengths in binned intervals of 50 amino acids. (**F**) Distribution of protein-to-nucleic acid length ratios (number of protein amino acids/number of nucleotides) across complexes in MODNAP. (**G**) Total count of annotated functions of nucleic acid or nucleic acid–protein complex in the MODNAP benchmark set. For nucleic acid–protein complex entries, the functional annotation of the protein is provided. The percentage of entries in MODNAP with each annotated function is noted. The number of PDB entries (*n*) for each type of nucleic acid or nucleic acid–protein complex is provided (total *n* = 1 039).

Motivated to probe how AlphaFold 3 performs on structures not explicitly seen during training and calibration, we also generated the MODNAP test set, which refers to the subset of 103 structures excluded from the training data. The statistics of the MODNAP test set (post-cutoff) showed a distribution of features broadly representative of the pre-cutoff entries (Supplementary Fig. S9). Together, the features of MODNAP suggest it serves as a representative dataset for benchmarking the modeling modified nucleic acids. First, MODNAP samples a range of nucleic acid functions and modification types, partially sampling the chemical and structural landscape. Second, it includes structures deposited after the AlphaFold 3 training cutoff, enabling evaluation on potentially unseen structures. One obvious caveat is that MODNAP does not fully represent the breadth of the natural and synthetic modified nucleotide chemical space. Nor does it sample the entire nucleic acid and nucleic acid–protein complex structure space. However, it nonetheless provides a starting point for systematically evaluating the modeling of modified nucleotide and nucleotide–protein complexes.

### Chemical and intramolecular validity of AlphaFold 3-generated models of modified nucleic acids

The ability of AlphaFold 3 to model the MODNAP benchmark set was evaluated to provide the scientific community with guidance on its performance and potential limitations in modeling modified nucleic acids and nucleic acid–protein complexes. AlphaFold 3 was selected for several reasons. First, it is among the most widely used and validated structure prediction tools available, owing to its high accuracy and the broad recognition brought by the 2024 Nobel Prize in Chemistry awarded for its development. It has demonstrated excellent performance across unmodified nucleic acid and nucleic acid–protein modeling tasks [55–57, 89, 90]. Second, AlphaFold 3 is one of a limited number of structure prediction platforms capable of modeling modified nucleic acids directly, leveraging CCDs to represent non-canonical residues. Many other available tools that support modified nucleic acid modeling rely on similar underlying algorithms or architectures, making AlphaFold 3 broadly representative of this class of methods. Third, AlphaFold 3 natively supports modeling of complex, multi-component assemblies, including nucleic acid–protein complexes, within a single unified framework, which is directly relevant to the heterogeneous PDB entries in the MODNAP benchmark set. Finally, AlphaFold 3 is actively maintained and updated by its developers, ensuring the model reflects current best practices in structure prediction. Beyond structure prediction, the rigorous assessment of modeled nucleic acids and nucleic acid–protein complex structures has long been a central component of community-wide efforts, such as RNA-Puzzles and CASP [91–93], where evaluations extend beyond global backbone structure accuracy to encompass stereochemical, geometric, and topological validity. Consistent with these efforts, previous studies have suggested that AI-based methods may generate chemically and physically implausible structures with chirality errors, stereochemical violations, hallucinations, entanglements, knots, and steric clashes [49, 78, 81, 94, 95]. However, whether AlphaFold 3 generates valid poses for modified nucleotides has not yet been systematically evaluated.

AlphaFold 3-generated models of MODNAP entries were first examined for whether they passed validity checks implemented in the PoseBusters test suite [78]. A model must pass several chemical and intramolecular validity checks to be considered PoseBusters test suite valid (referred to as PB-valid) [78]. More than 97% of AlphaFold 3-generated modified nucleic acid and nucleic acid–protein complex models passed chemical validity checks, including successful molecular loading into the PoseBusters toolkit (mol_pred_loaded), RDKit sanitization of chemical structures (sanitization), conversion to valid standard InChI representations (inchi_convertible), and the absence of radical species such as atoms with unpaired electrons (no_radicals) (Fig. 3A–C). A small number of models (1.8%) failed sanitization and InChI convertibility checks due to incorrect bonding and geometry in the phosphate group (Fig. 3A–C). Apart from CCD IDs C49 (4-thio, 5-fluoro, 5-methyl-2′-deoxycytidine 5′-monophosphate, passing rate 38%) and UCL (5-chloro-2′-deoxyuridine 5′-dihydrogen phosphate, passing rate 57%), models that failed chemical validity checks did not appear to cluster around specific CCD IDs (Supplementary Fig. S10). Additional CCDs failed sanitation tests, but each was represented by only a single PDB entry, making it difficult to determine whether these failures reflect a broader trend. Most CCD IDs associated with failed cases also contained models that passed, suggesting that chemical validity issues are not driven by particular chemical components or residue types but rather seem to arise sporadically.

**Figure 3.**
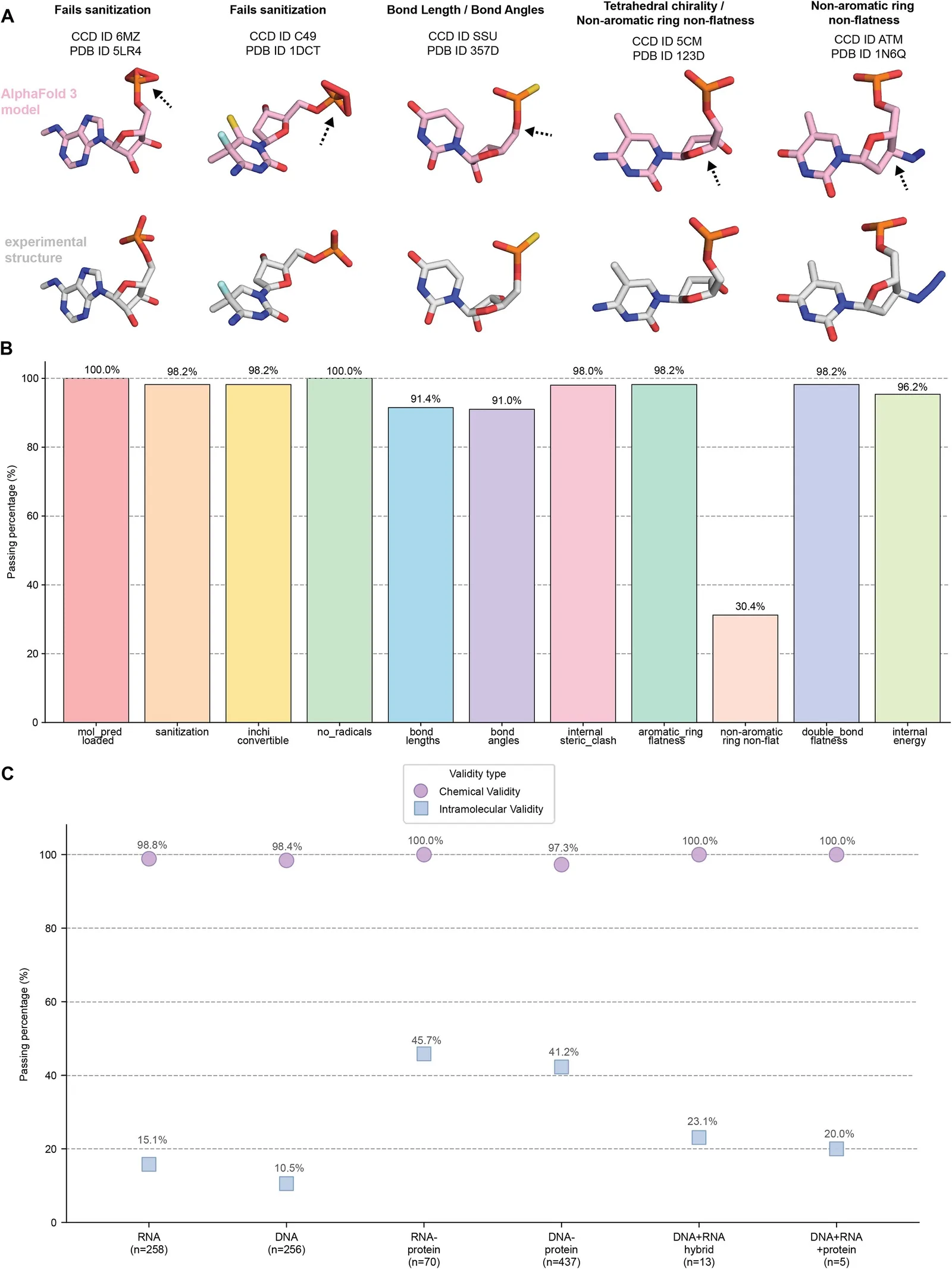
PoseBusters test suite validity of modified nucleotides in AlphaFold 3-generated models of modified nucleic acids and nucleic acid–protein complexes. (**A**) Examples of AlphaFold 3 models of modified nucleotides not considered PB-valid. Modified nucleotides from AlphaFold 3 models and experimental structures have carbon atoms colored pink and gray, respectively. Models that fail sanitation checks frequently exhibit hallucinations in the phosphate group. (**B**) Passing percentage of modified nucleotides in AlphaFold 3 models of all MODNAP entries as a function of each PoseBusters test-suite descriptors of chemical validity and intramolecular validity. (**C**) Passing percentage of chemical validity and intramolecular validity terms for modified nucleotides in AlphaFold 3 models of all MODNAP entries as a function of molecule type.

Apart from failures arising from violations of the non-aromatic ring non-flat criteria, >91% of AlphaFold 3-generated nucleic acid and nucleic acid–protein complex models passed intramolecular validity checks (Fig. 3A and B). A small number of models exhibited local geometric issues, including deviations in bond lengths, bond angles, or internal energy. However, only 30.4% of models passed the PoseBusters non-aromatic ring non-flat test, suggesting that ribose moieties frequently deviate from expected ring planarity and exhibit non-ideal puckering or geometric distortion (Fig. 3A and B). The percentage of models passing intramolecular validity checks including the non-aromatic ring non-flatness criterion varied across molecule types (Fig. 3C). Low pass rates were observed for RNA (15.1%) and DNA (10.5%) molecules, whereas higher pass rates were found for RNA–protein (45.7%) and DNA–protein complexes (41.2%). This trend suggests that co-folding in the presence of protein partners may improve overall intramolecular geometric consistency of the generated nucleic acid structures. In contrast, the natural (unmodified) nucleotides present within the same AlphaFold 3 models passed the PoseBusters intramolecular validity tests at nearly 100% for all evaluated criteria, including the non-aromatic ring non-flatness test (Supplementary Fig. S11), indicating that these geometric violations are largely associated with modified nucleotides rather than with canonical ribose or deoxyribose residues. Together, these results highlight the importance of evaluating the chemical and intramolecular validity of AlphaFold 3-generated modified nucleotide structures prior to downstream analyses, as such quality checks are essential for reliably assessing the integrity of predicted modified nucleic acids and nucleic acid–protein complex models.

### Entanglements in AlphaFold 3 models of modified RNA structures

Predicted RNA structures, especially those with complex topological folds, have been reported to contain entanglements, defined as an arrangement in which one base paired structural element, such as a loop or duplex, is threaded by another structural element, such as a single strand [96]. Entanglements of AlphaFold 3 models of modified RNA, RNA–protein complexes, and DNA–RNA hybrids of MODNAP entries were identified with RNAspider [80, 81]. Entanglements were detected in models across several CCD IDs with the number of affected models varying considerably between groups, from single instances (i.e. 2AU and 5BU) to multiple instances (i.e. A2M, 3DA, A23, and CCC) (Supplementary Fig. S12A and B). Both lasso- and interlace-type entanglements were identified. As representative examples, AlphaFold 3 models containing CCD IDs A2M or A23 each exhibited an L(S) type lasso entanglement, in which a loop was threaded through a downstream single-stranded segment (Supplementary Fig. S13). In contrast, an AlphaFold 3 model containing CCD ID CCC showed no detected entanglement (Supplementary Fig. S13). AlphaFold 3 models containing CCD IDs that most frequently exhibited entanglements, including CCC, CBV, A23, A2M, and 1MA, corresponded predominantly to complex ribozyme and riboswitch folds (Supplementary Fig. S14). This observation is consistent with previous reports by Popenda *et al*. showing that highly structured, multidomain RNA architectures found in ribozymes and riboswitch folds are particularly susceptible to entanglements [81]. Together, the analysis suggests that the tendency of AlphaFold 3 to introduce entanglements is not necessarily due to the presence of a specific modified nucleotide but is instead dependent on the RNA tertiary fold itself, and is concentrated in structurally intricate classes already known to adopt topologically entangled folds.

### Performance of AlphaFold 3 on the MODNAP benchmark set

The performance of AlphaFold 3 on the MODNAP benchmark set is described below with results stratified by both molecule type and modification type. Toward comparison of AlphaFold 3-generated models with ground truth experimental structures, we first used RMSD [97], a widely used metric for structural superposition of nucleic acids and nucleic acid–protein complexes [47, 49, 55, 98]. AlphaFold 3 achieves high success rates across all different molecule types when evaluated at a 2.5-Å RMSD threshold with RNA–protein complexes performing best (97.1%), followed by DNA–RNA hybrids (92.3%), DNA (92.2%), DNA–protein complexes (90.4%), RNA (88.4%), and DNA–RNA–protein complexes (80%) (Fig. 4A and Table 1). Across different modification types, success rates at this threshold were likewise uniformly high, ranging from ∼90% for base and sugar modifications to 100% for phosphate, base+sugar, and base+phosphate modifications (Supplementary Fig. S15A and Table 1). When the RMSD threshold was relaxed to 3 Å, only minimal increases in success rates were observed across most categories by molecule type or modification type (Fig. 4A and B, and Supplementary Fig. S15A and B). An exception was DNA–RNA–protein complexes, which contained only five structures and exhibited a larger proportion of predictions within the 2.0–2.5 Å RMSD range (Fig. 4A). PB-valid success rates, which reflect predictions passing all chemical and intramolecular validity criteria, were substantially lower than raw RMSD-based success rates across all categories (Figs 3A–C and 4A, and Supplementary Fig. S15A). DNA–protein (41.2%) and RNA–protein (45.7%) complexes showed the highest PB-valid rates, whereas DNA structures showed the lowest (10.5%). By modification type, PB-valid rates followed a similar overall pattern of substantially reduced validity relative to RMSD-based success, spanning a 17.6%–50% range with base+sugar modifications the lowest (17.6%). These results suggest that although AlphaFold 3 frequently generates nucleic acid structures that match the experimental reference, a substantial fraction of predictions contain non-idealized structures at the modified nucleotides.

**Figure 4.**
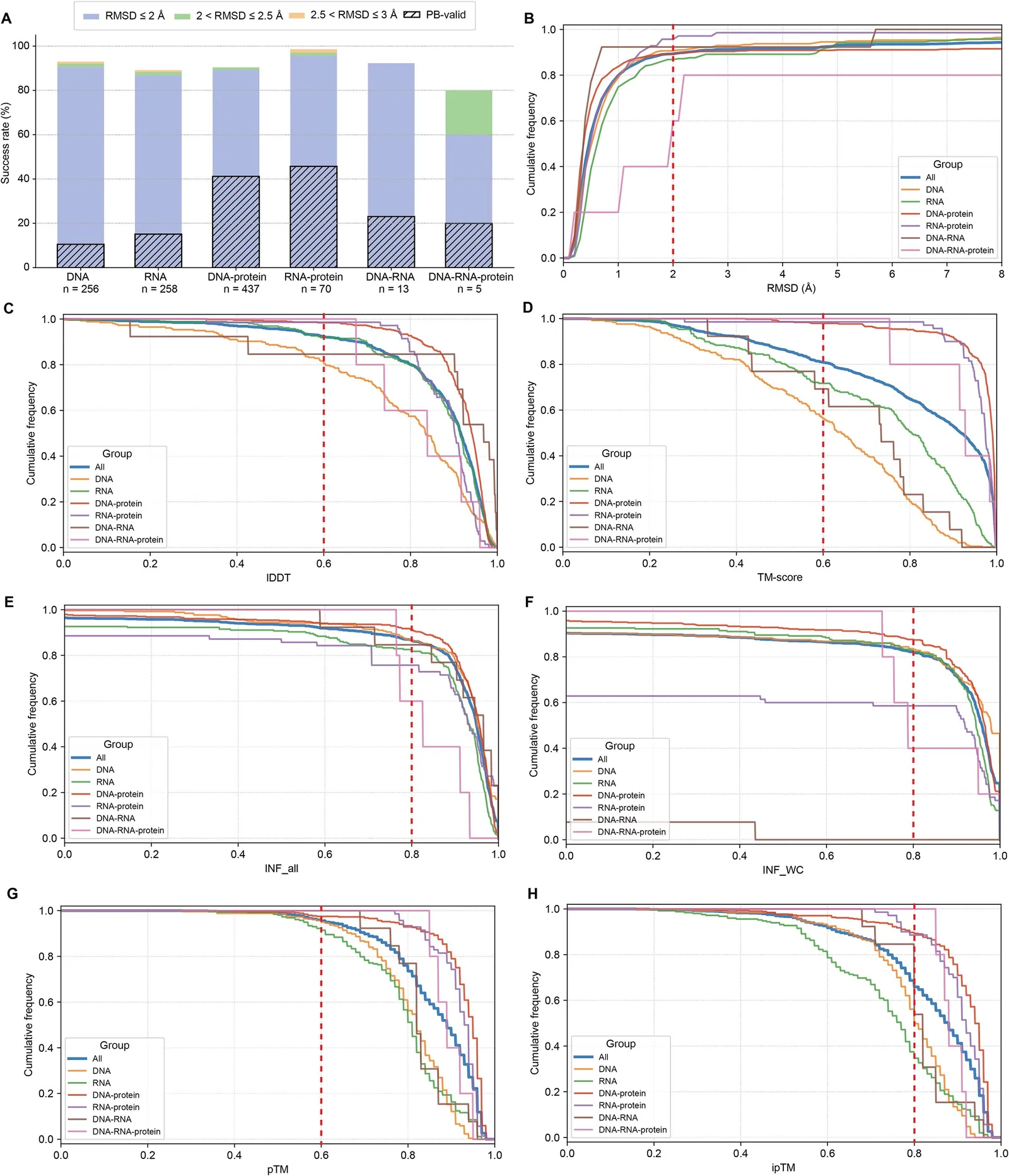
Performance of AlphaFold 3 on the MODNAP benchmark set by molecule type. (**A**) Bar plot of the success rates under RMSD threshold values of 2, 2.5, or 3 Å, and PB-valid. Success rates for RMSD thresholds are defined as the percentages of predictions under the specific RMSD threshold values. Success rates for PB-valid cutoff are defined as the percentages of predictions that passed each chemical validity and intramolecular validity tests and were labeled in dark shades. Cumulative frequency for (**B**) RMSD with a threshold value of 8 Å, (**C**) lDDT with a threshold value of 1, (**D**) TM-score with a threshold value of 1, (**E**) INF_all values with a maximum value of 1, (**F**) INF_WC values with a maximum value of 1, (**G**) pTM values with a threshold value of 1, and (**H**) ipTM values with a threshold value of 1. The dotted red lines represent standard threshold values for modeling accuracy.

**Table 1.** AlphaFold 3 prediction success rates for nucleic acid structures containing modified nucleotides, stratified by molecule type or modification type

Molecule type	MODNAP % success rate (RMSD < 2.5 Å)	% PB-Valid
DNA (*n* = 256)	92.2	10.5
RNA (*n* = 258)	88.4	15.1
DNA–protein (*n* = 437)	90.4	41.2
RNA–protein (n = 70)	97.1	45.7
DNA–RNA (*n* = 13)	92.3	23.1
DNA–RNA–protein (*n* = 5)	80.0	20.0
**Modification type**	**MODNAP** **% Success rate** **(RMSD < 2.5 Å)**	**% PB-Valid**
Base (*n* = 696)	89.9	26.6
Sugar (*n* = 290)	91.0	27.9
Phosphate (*n* = 34)	100.0	35.3
Base+sugar (*n* = 17)	100.0	17.6
Base+phosphate (*n* = 2)	100.0	50.0

Success rate is defined as the percentage of predicted poses with RMSD <2.5 Å relative to the experimental structure. PB-valid rate is the percentage of poses passing all PoseBusters test suite quality checks, independent of RMSD. *n* denotes the number of structures in each category.

Since RMSD can be insensitive to local errors and may not reliably distinguish partially correct models from incorrect ones, we also compared predicted and experimental structures using other widely used structural similarity metrics (lDDT, TM-score, INF_all, INF_WC, I-INF). Across all structural similarity metrics, AlphaFold 3 models of MODNAP entries achieved consistently high accuracy across different molecule types and modification types. lDDT scores were high across most groups with the majority of predictions exceeding 0.8 (Fig. 4C and Supplementary Fig. S15C). At the 0.6 lDDT threshold commonly used to indicate a likely accurate prediction, ∼95% of models across all categories met the threshold. DNA and DNA–RNA–protein complexes or molecules with phosphate modifications showed cumulative distributions shifted modestly toward lower values relative to the other categories, indicating a somewhat higher proportion of predictions with reduced local structural accuracy. Similarly, TM-score values were generally high across categories, though with greater spread than lDDT (Fig. 4D and Supplementary Fig. S15D). At the 0.6 threshold commonly used to indicate a likely accurate prediction, approximately over 90% of DNA–protein and RNA–protein predictions met this threshold, compared to 60%–80% for DNA, RNA, and DNA–RNA hybrids. By modification type, the spread at this threshold was narrower, with 78%–90% of base, sugar, phosphate, and base+sugar predictions meeting threshold. INF_all and INF_WC scores were generally high for DNA, RNA, and DNA–protein groups, but RNA–protein complexes showed notably lower INF_WC scores, with a substantial fraction of predictions failing to recover Watson–Crick base pairing interactions (Fig. 4E and F). A similar pattern was observed by modification type: INF_all scores were high and comparable across categories, whereas phosphate modifications showed notably lower INF_WC scores relative to other modification types (Supplementary Fig. S15E and F). To directly assess how well AlphaFold 3 captures interactions between components, we calculated the Interaction INF (I-INF) for each interface [77]. DNA, RNA, DNA–protein, and DNA–RNA–protein interfaces mostly reached the I-INF ≥ 0.8 threshold, while RNA–protein interfaces did so less consistently, with just over half of predictions reaching this threshold (Supplementary Fig. S16A). DNA–RNA interfaces performed worst, though this group was limited by a small sample size. By modification type, base, sugar, base+sugar, and base+phosphate modifications mostly reached the I-INF ≥ 0.8 threshold, while phosphate modifications did so less consistently, with just under 80% of predictions reaching this threshold (Supplementary Fig. S16B). Overall, these results indicate that AlphaFold 3 models of modified nucleic acid complexes accurately capture the interaction interfaces relative to native structures, with the main limitation being RNA–protein or DNA–RNA hybrid interfaces and, among modification types, phosphate modifications.

AlphaFold 3’s confidence metrics suggest mixed reliability in the model’s self-assessment. At the standard pTM > 0.6 threshold, the vast majority of predictions across all categories exceeded this threshold (Fig. 4G), suggesting that AlphaFold 3 itself considers the overall predicted structure likely to match the ground truth. However, at the standard ipTM > 0.8 threshold for confident interface prediction, complexes containing protein (DNA–protein, RNA–protein, and DNA–RNA–protein) showed high confidence, with over 80% of predictions meeting this threshold (Fig. 4H). In contrast, fewer than 60% of DNA, RNA, and DNA–RNA predictions met this threshold, with RNA complexes showing particularly low ipTM values, indicating that AlphaFold 3 itself has limited confidence in the predicted interfaces for complexes lacking protein. By modification type, ipTM values did not separate as clearly, with most categories (base, sugar, phosphate) clustering around 60%–65% at this threshold, below the two small multi-modification categories (base+sugar, base+phosphate) (Supplementary Fig. S15G and H). Together, these data suggest that AlphaFold 3 predicts structures of modified nucleic acids and their complexes with generally high global and local accuracy across most categories, but that AlphaFold 3’s performance and confidence in predicted interfaces is lower for nucleic acids lacking bound protein, and, among modification types, for nucleic acids bearing phosphate modifications.

### Comparison of ground truth experimental structures with AlphaFold 3 models

Representative structures demonstrate that AlphaFold 3 generally produces accurate predictions across a broad range of nucleic acid and nucleic acid–protein complexes containing modified nucleotides, spanning DNA (Fig. 5), RNA (Fig. 6), DNA–protein complexes (Fig. 7), RNA–protein complexes (Fig. 8), and DNA–RNA hybrids (Fig. 9). AlphaFold 3 models superimpose well onto experimental structures, with RMSD values for the modified nucleotide and its immediate context typically below 2 Å, and global fold metrics (TM-score, lDDT, pTM, ipTM, INF_all) frequently exceeding 0.6. This indicates that AlphaFold 3 captures not only the correct overall fold and, where applicable, the nucleic acid–protein interface geometry, but also the local placement of modified functional groups, such as methylated (5MC, OMG, OMC, OMU), halogenated (BRU, 5IU, 5BU), thiophosphate-containing (AS, G46, S6G), and other bulkier base or backbone modifications (2NT, ZDU, UMS, FHU, M2G), in proper register with the surrounding bases and nucleic acid backbone. OMG, BRU, 5IU, and 5BU are among the 12 most common CCD IDs found in structures present in the MODNAP dataset.

**Figure 5.**
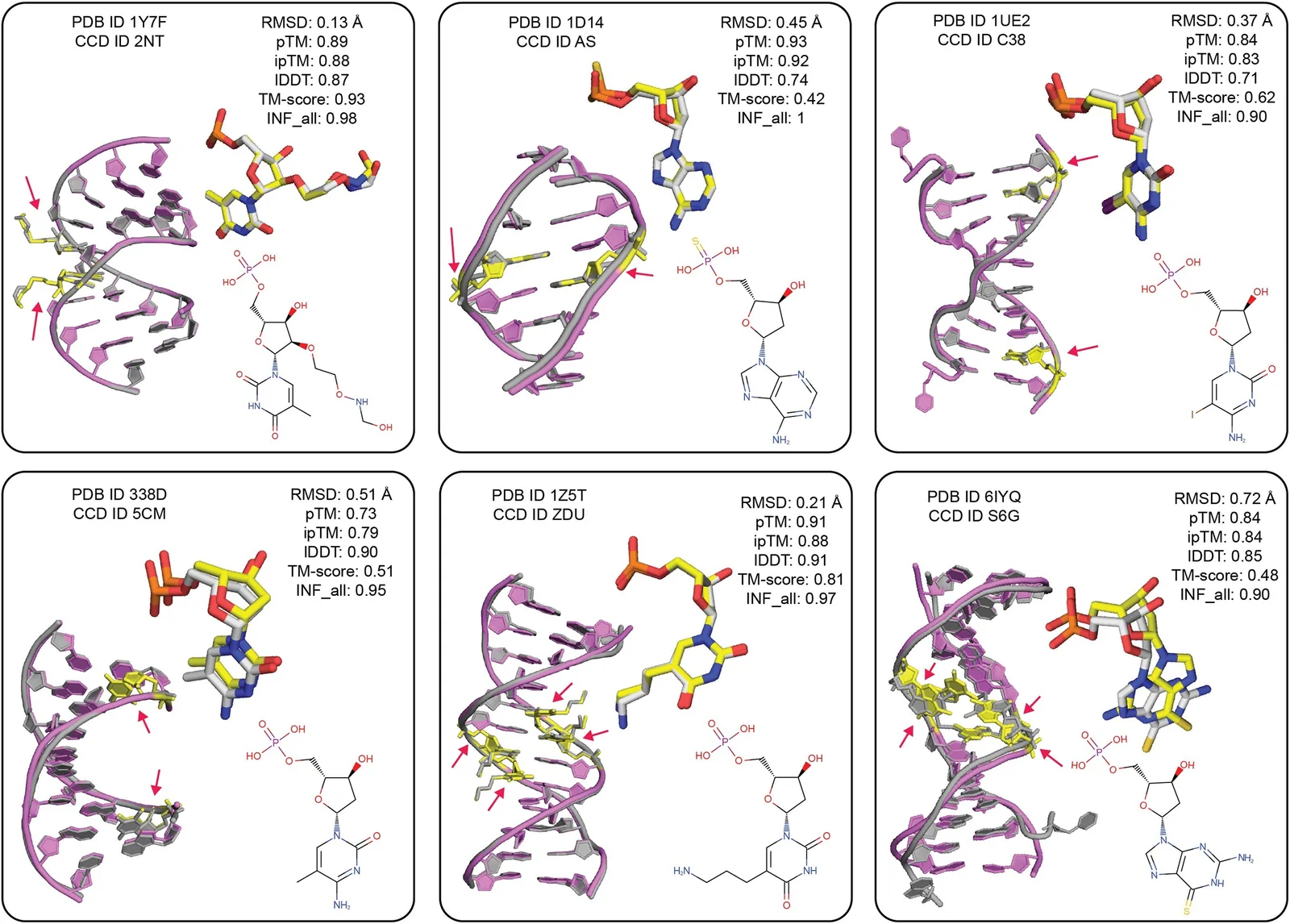
AlphaFold 3 models of modified DNA molecules in the MODNAP benchmark set. Overlay of the ground truth experimental DNA structure (gray) and the AlphaFold 3-predicted DNA structure (purple), with modified nucleotide(s) shown in yellow and highlighted by red arrows. An overlay of the experimental (gray sticks) and AlphaFold 3-predicted (yellow sticks) modified nucleotide, extracted from the corresponding structures, is also shown. The chemical structure of the modified nucleotide is provided. Structure comparison metrics (RMSD, lDDT, TM-score, and INF_all) and AlphaFold 3 confidence metrics (pTM and ipTM) are indicated. Examples shown: DNA with 2′-O-[2-[hydroxy(methyleneamino)oxy]ethyl] thymidine (CCD ID 2NT in PDB ID 1Y7F) [99], DNA with 2-deoxy-adenosine-5′-thio-monophosphate (CCD ID AS in PDB 1D14) [100], DNA with 5-iodo-2′-deoxycytidine-5′-monophosphate (CCD ID C38 in PDB ID 1UE2) [101], DNA with 5-methyl-2′-deoxycytidine 5′-monophosphate (CCD ID 5CM in PDB ID 338D), DNA with 5-(3-aminopropyl)-2′-deoxyuridine (CCD ID ZDU in PDB ID 1Z5T) [102], and DNA cross-linked with 6-thioguanine-6-thioguanine (CCD ID S6G in PDB ID 6IYQ) [103].

**Figure 6.**
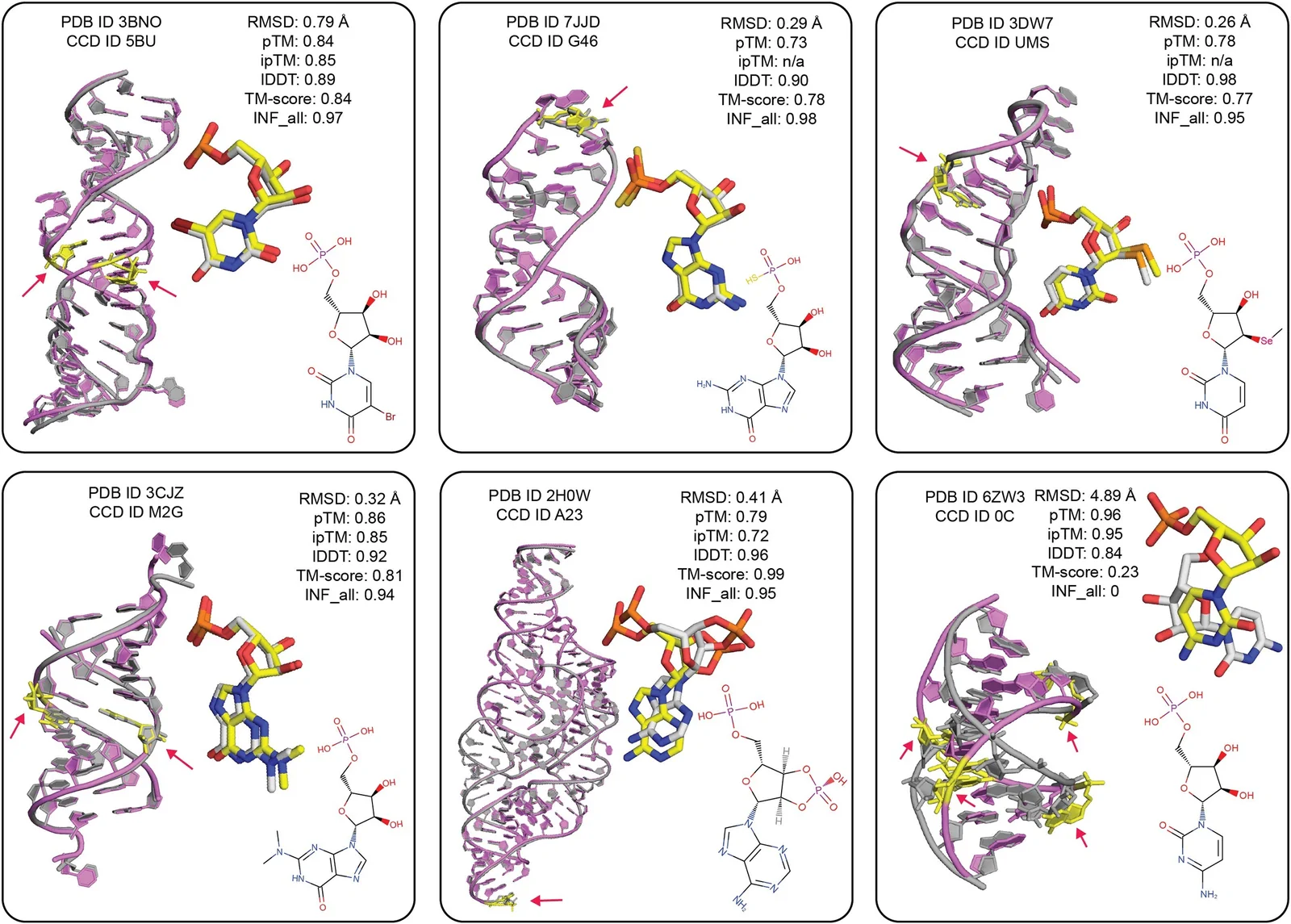
AlphaFold 3 models of modified RNA molecules in the MODNAP benchmark set. Overlay of the ground truth experimental RNA structure (gray) and the AlphaFold 3-predicted RNA structure (purple), with modified nucleotide(s) shown in yellow and highlighted by red arrows. An overlay of the experimental (gray sticks) and AlphaFold 3-predicted (yellow sticks) modified nucleotide, extracted from the corresponding structures, is also shown. The chemical structure of the modified nucleotide is provided. Structure comparison metrics (RMSD, lDDT, TM-score, and INF_all) and AlphaFold 3 confidence metrics (pTM and ipTM) are indicated. Examples shown: RNA with 5-bromouridine-5′-monophosphate (CCD ID 5BU in PDB ID 3BNO) [104], RNA with guanosine 5′-monothiophosphate (CCD ID G46 in PDB ID 7JJD) [105], RNA with 2′-methylselenyl-2′-deoxyuridine-5′-phosphate (CCD ID UMS in PDB ID 3DW7) [106], RNA with N2-dimethylguanosine 5′-monophosphate (CCD ID M2G in PDB ID 3CJZ) [107], RNA with adenosine-5′-phosphate-2′, 3′-cyclic phosphate (CCD ID A23 in PDB ID 2H0W) [108], and RNA with L-cytidine-5′-monophosphate (CCD ID 0C in PDB ID 6ZW3) [109].

**Figure 7.**
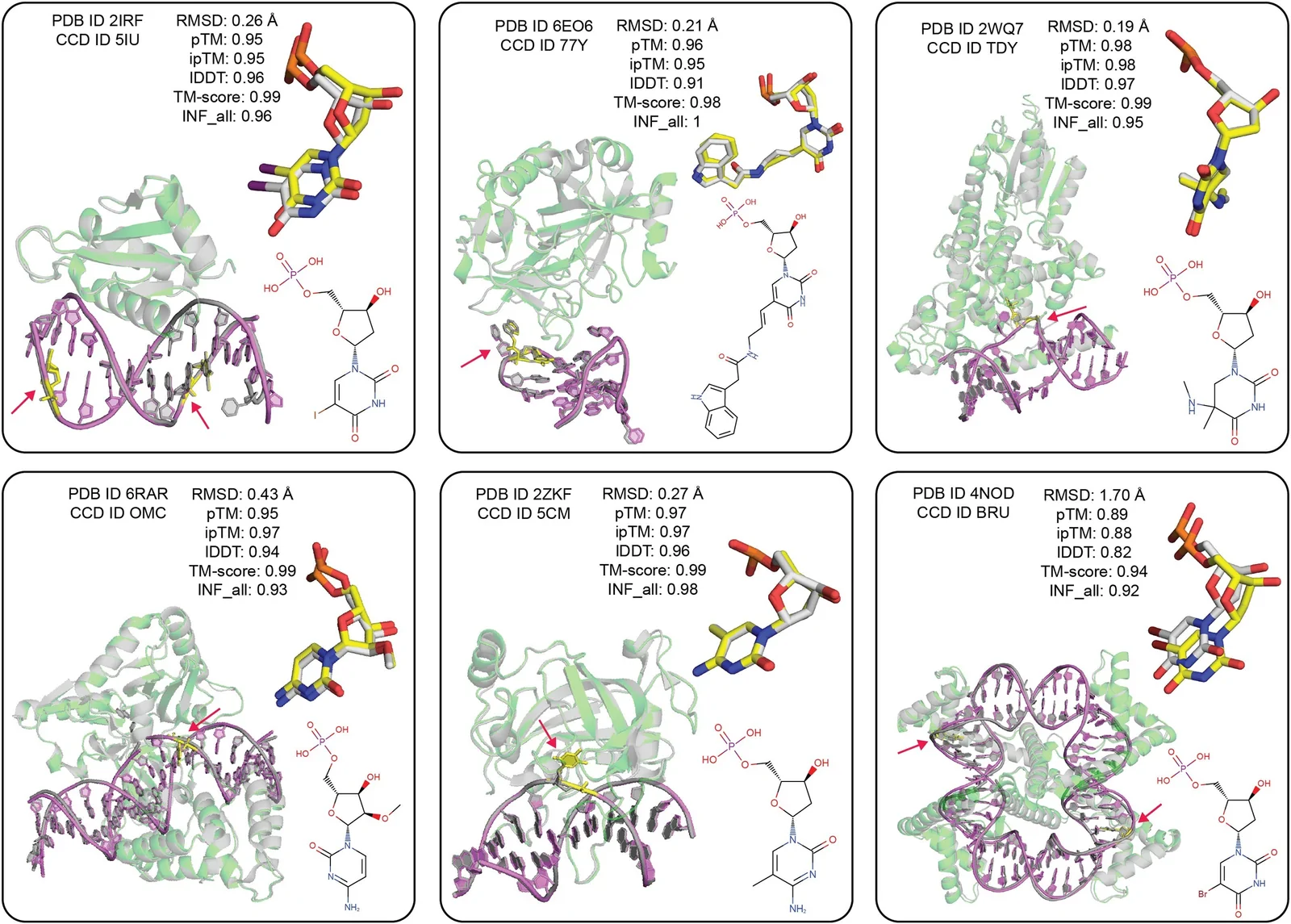
AlphaFold 3 models of modified DNA–protein complexes in the MODNAP benchmark set. Overlay of the ground truth experimental DNA–protein complex structures (gray) and the AlphaFold 3-predicted DNA–protein complex structure (purple DNA, green protein), with modified nucleotide(s) shown in yellow and highlighted by red arrows. An overlay of the experimental (gray sticks) and AlphaFold 3-predicted (yellow sticks) modified nucleotide, extracted from the corresponding structures, is also shown. The chemical structure of the modified nucleotide is provided. Structure comparison metrics (RMSD, lDDT, TM-score, and INF_all) and AlphaFold 3 confidence metrics (pTM and ipTM) are indicated. Examples shown: DNA–protein complex with 5-iodo-2′-deoxyuridine-5′-monophosphate (CCD ID 5IU in PDB ID 2IRF) [110], DNA–protein complex with 5-(3-(2-(1H-indol-3-yl)acetamide-N-yl)-1-propen-1-yl)-2′-deoxyuridine (CCD ID 77Y in PDB ID 6EO6) [111], DNA–protein complex with 5-(methylamino)thymidine 5′-(dihydrogen phosphate) (CCD ID TDY in PDB ID 2WQ7) [112], DNA–protein complex with 2′-O-methylcytidine 5′-monophosphate (CCD ID OMC in PDB ID 6RAR) [113], DNA–protein complex with 5-methyl-2′-deoxycytidine 5′-monophosphate (CCD ID 5CM in PDB ID 2ZKF) [114], and DNA–protein complex with 5-bromo-2′-deoxyuridine-5′-monophosphate (CCD ID BRU in PDB ID 4NOD) [115].

**Figure 8.**
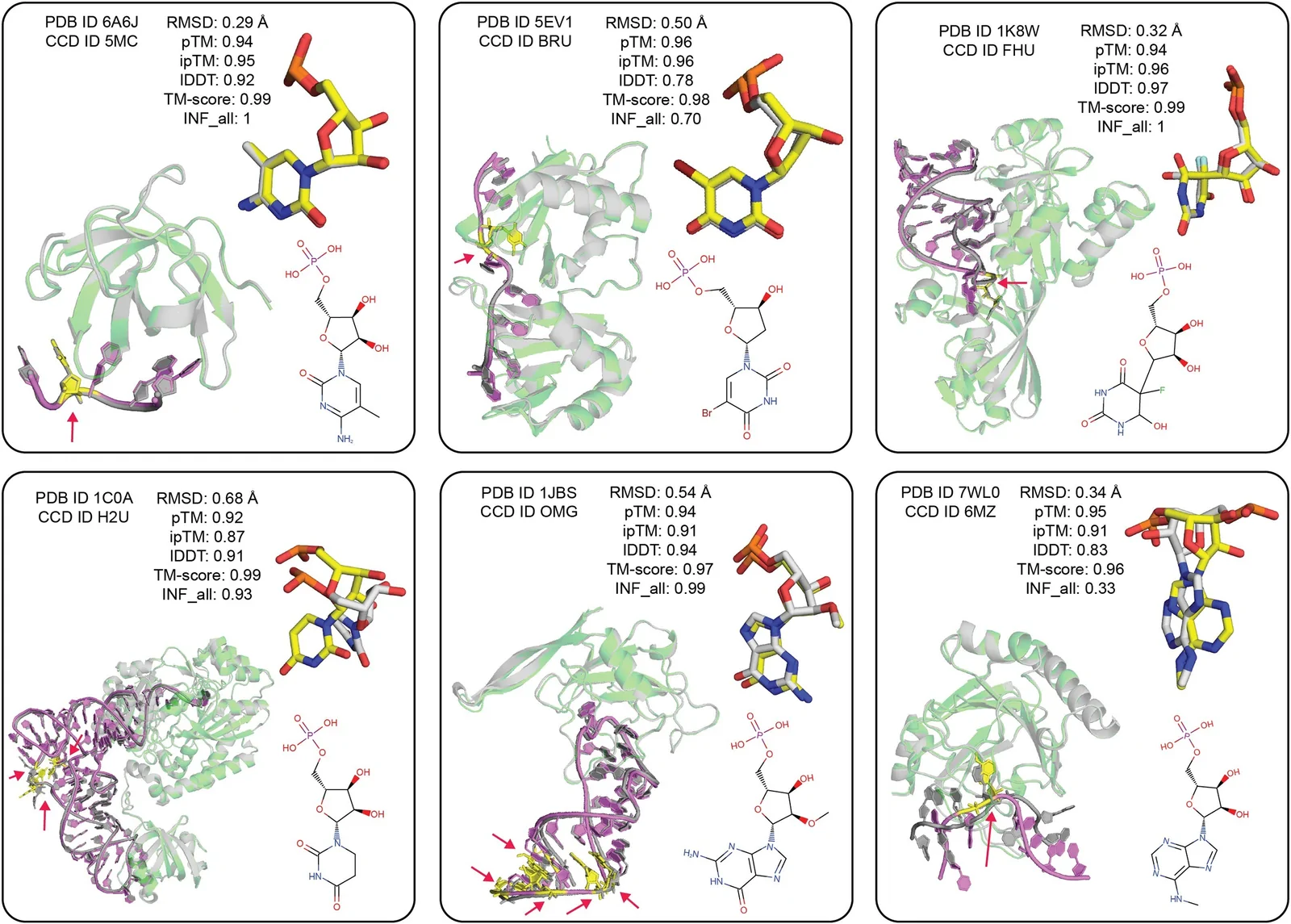
AlphaFold 3 models of modified RNA–protein complexes in the MODNAP benchmark set. Overlay of the ground truth experimental RNA–protein complex structures (gray) and the AlphaFold 3-predicted RNA–protein complex structure (purple RNA, green protein), with modified nucleotide(s) shown in yellow and highlighted by red arrows. An overlay of the experimental (gray sticks) and AlphaFold 3-predicted (yellow sticks) modified nucleotide, extracted from the corresponding structures, is also shown. The chemical structure of the modified nucleotide is provided. Structure comparison metrics (RMSD, lDDT, TM-score, and INF_all) and AlphaFold 3 confidence metrics (pTM and ipTM) are indicated. Examples shown: RNA–protein complex with 5-methylcytidine-5′-monophosphate (CCD ID 5MC in PDB ID 6A6J) [116], RNA–protein complex with 5-bromo-2′-deoxyuridine-5′-monophosphate (CCD ID BRU in PDB ID 5EV1) [117], RNA–protein complex with (5S, 6R)-5-fluoro-6-hydroxy-pseudouridine-5′-monophosphate (CCD ID FHU in PDB ID 1K8W) [118], RNA–protein complex with 5, 6-dihydrouridine-5′-monophosphate (CCD ID H2U in PDB ID 1C0A) [119], RNA–protein complex with O2'-methylguanosine-5′-monophosphate (CCD ID OMG in PDB ID 1JBS) [120], and RNA–protein complex with N6-methyladenosine 5′-monophosphate (CCD ID 6MZ in PDB ID 7WL0) [121].

**Figure 9.**
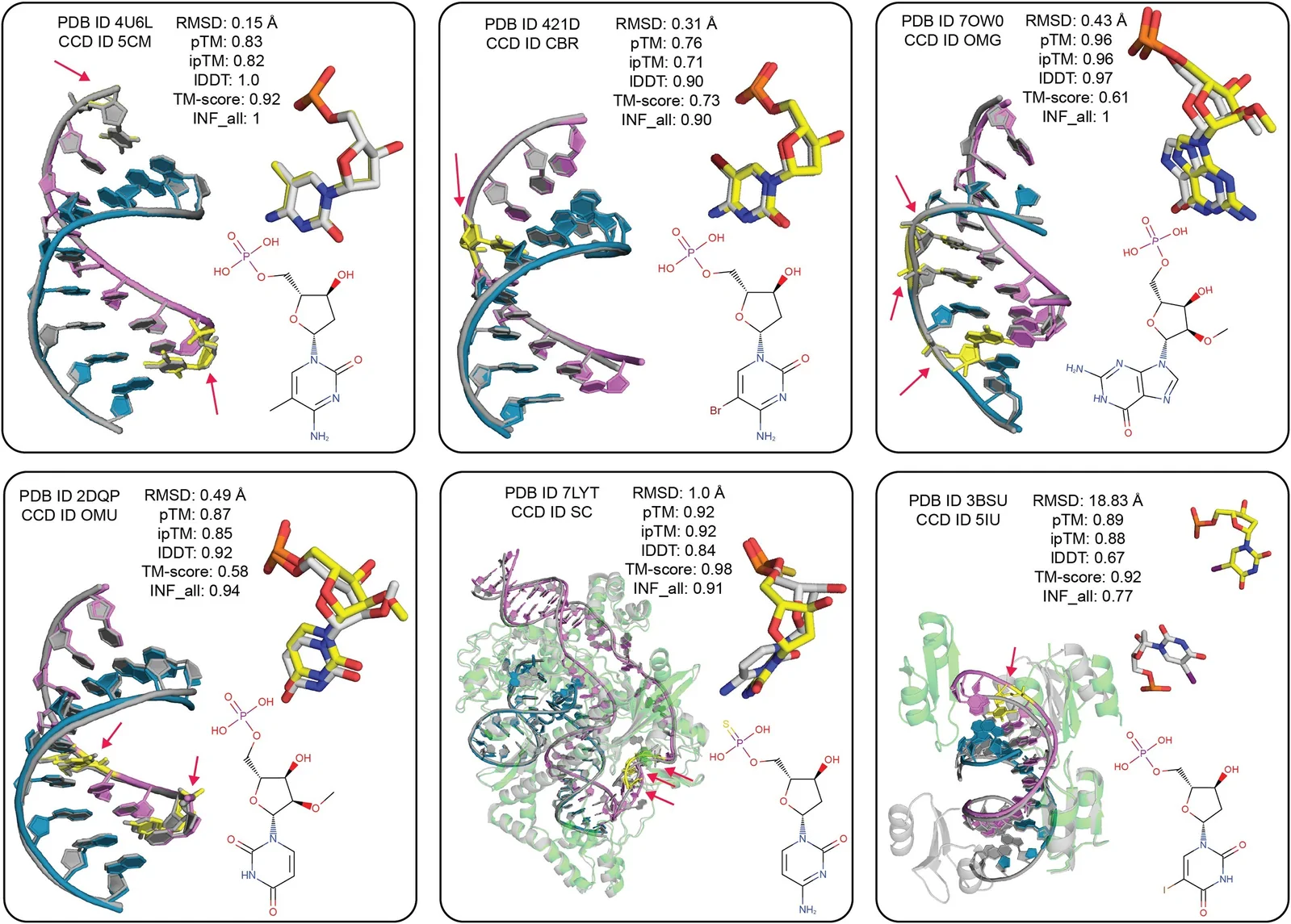
AlphaFold 3 models of modified DNA–RNA hybrids in the MODNAP benchmark set. Overlay of the ground truth experimental DNA–RNA hybrid structures (gray) and the AlphaFold 3-predicted RNA–protein complex structure (purple DNA, blue RNA, green protein), with modified nucleotide(s) shown in yellow and highlighted by red arrows. An overlay of the experimental (gray sticks) and AlphaFold 3-predicted (yellow sticks) modified nucleotide, extracted from the corresponding structures, is also shown. The chemical structure of the modified nucleotide is provided. Structure comparison metrics (RMSD, lDDT, TM-score, and INF_all) and AlphaFold 3 confidence metrics (pTM and ipTM) are indicated. Examples shown: DNA–RNA hybrid with 5-methyl-2′-deoxycytidine 5′-monophosphate (CCD ID 5CM in PDB ID 4U6L) [122], DNA–RNA hybrid with 5-bromo-2′-deoxycytidine-5′-monophosphate (CCD ID CBR in PDB ID 421D) [123], DNA–RNA hybrid with O2'-methylguanosine-5′-monophosphate (CCD ID OMG in PDB ID 7OW0) [124], DNA–RNA hybrid with O2'-methyluridine 5′-monophosphate (CCD ID OMU in PDB ID 2DQP) [125], DNA–RNA hybrid with 2-deoxy-cytidine-5′-thiophosphorate (CCD ID SC in PDB ID 7LYT) [126], and DNA–RNA hybrid with 5-iodo-2′-deoxyuridine-5′-monophosphate (CCD ID 5IU in PDB ID 3BSU) [127].

A subset of cases show substantially larger deviations, including PDB 6ZW3 (RMSD 4.89 Å, TM-score 0.23, INF_all 0) and PDB 3BSU (RMSD 18.83 Å, INF_all 0.77), where local misplacement of the modified nucleotide and distortion of the surrounding helix are observed (red arrows). Lower-accuracy predictions were most frequently observed for structures containing extensive nucleotide modification, particularly those in which the majority of nucleobases were modified, as well as for RNA and DNA molecules with complex tertiary architectures (i.e. ribozymes and riboswitches) and large nucleic acid–protein assemblies comprising more than three subunits. These findings suggest that prediction accuracy is influenced not only by the presence of modified nucleotides but also by overall structural complexity, including tertiary folding and multicomponent intermolecular interactions. These examples are illustrative rather than exhaustive and are not intended to represent the full distribution of model performance across the benchmark set.

### Placement of modified nucleotides versus full structure accuracy

When RMSD was calculated over the full structure, AlphaFold 3 achieved high accuracy across most molecule types showing >85% of predictions falling below 2.0 Å RMSD (Fig. 10A). The full structure accuracy held regardless of the type of modification as well: 88.8%–100% of structures fell below 2.0 Å RMSD when the modification involved the base, sugar, phosphate, or a combination (Supplementary Fig. S17A). When RMSD was instead calculated specifically over the modified nucleotide(s) only, accuracy decreased across every category relative to the full-structure metric. Comparing molecule types, the fraction of modified nucleotides below 2.0 Å RMSD fell to 65.2% for DNA, 58.5% for RNA, 70.3% for DNA–protein, 40% for DNA–RNA–protein complexes, and 48.6% for RNA–protein complexes, while DNA–RNA hybrids remained comparatively robust (92.3%, unchanged across thresholds) (Fig. 10B). A similar decline trend emerged when broken down by modification chemistry: base modifications were correctly placed below 2.0 Å RMSD in only 66.7% of cases and sugar modifications in 62.8%, while phosphate modifications fell to just 38.2%, the lowest accuracy of any category or modification type examined (Supplementary Fig. S17B). This systematic gap between full structure and modification-specific RMSD indicates that AlphaFold 3 reliably reconstructs the overall global topology of modified nucleic acid structures, but is comparatively less accurate at placing the fine atomic detail of the modification itself (see examples in Figs 5–9). This likely reflects both the relative scarcity of modified-nucleotide examples in the training data compared to canonical residues, and the fact that local errors in modified residue placement contributes proportionally little to whole-structure superposition metrics even when the modification itself is misplaced.

**Figure 10.**
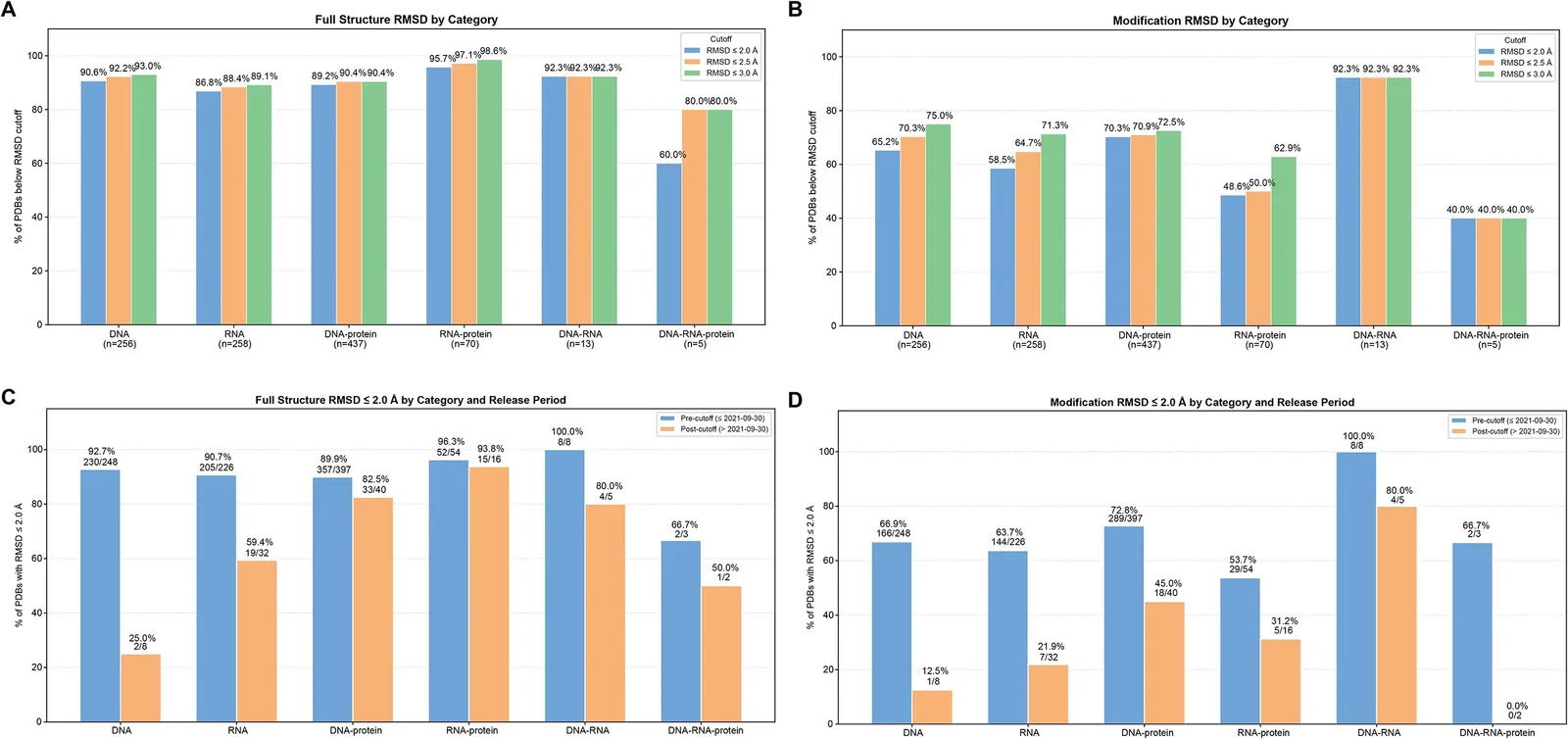
Performance of AlphaFold 3 on MODNAP entries pre- and post-training cutoff date by molecule type. (**A**) Percentage of PDB entries with full structure RMSD of AlphaFold 3 models versus experimental structures for all MODNAP entries separately under RMSD threshold values of 2, 2.5, or 3 Å. (**B**) Percentage of PDB entries with modified nucleotide RMSD of AlphaFold 3 models versus experimental structures for all MODNAP entries separately under RMSD threshold values of 2, 2.5, or 3 Å. (**C**) Percentage of PDB entries with full structure RMSD of AlphaFold 3 models under RMSD threshold values of 2 Å for MODNAP entries released before (blue, pre-cutoff) or after (orange, post-cutoff) AlphaFold 3’s training cutoff date. (**D**) Percentage of PDB entries with modified nucleotide RMSD of AlphaFold 3 models under RMSD threshold values of 2 Å for MODNAP entries released before (blue, pre-cutoff) or after (orange, post-cutoff) AlphaFold 3’s training cutoff date.

### Performance of AlphaFold 3 on the MODNAP test set

To assess whether AlphaFold 3’s performance reflects genuine generalization or partial memorization of structures present in its training set, we stratified the benchmark by PDB release date relative to AlphaFold 3’s training cutoff. For full structure RMSD ≤ 2.0 Å, performance was consistently lower for post-cutoff structures than pre-cutoff structures across nearly every category. Across molecule types, DNA dropped from 92.7% to 25.0% post-cutoff, RNA from 90.7% to 59.4%, DNA–protein complexes from 89.9% to 82.5%, RNA–protein complexes from 96.3% to 93.8%, DNA–RNA hybrids from 100.0% to 80.0%, and DNA–RNA–protein hybrids from 66.7% to 50.0% (Fig. 10C). Across modification type, base dropped from 91.3% to 68% post-cutoff, sugar from 90.3% to 66.7%, phosphate from 94.4% to 93.8%; and base+sugar and base+phosphate lacked post-cutoff structures present to analyze (Supplementary Fig. S17C). The decline in performance for post-cutoff structures with RMSD ≤ 2.0 Å was even more pronounced when evaluating the local RMSD value restricted to the modified nucleotide residue alone. For modification type, DNA fell from 66.9% to 12.5%, RNA from 63.7% to 21.9%, DNA–protein complexes from 72.8% to 45.0%, RNA–protein complexes from 53.7% to 31.2%, DNA–RNA hybrid from 100.0% to 80.0%, and DNA–RNA–protein hybrids from 66.7% to 0.0% (Fig. 10D). Across modification type, base dropped from 70% to 38.7% post-cutoff, sugar from 64.4% to 25%, phosphate from 55.6% to 18.8%; and base+sugar and base+phosphate lacked post-cutoff structures present to analyze (Supplementary Fig. S17D). Together, these results suggest that a portion of AlphaFold 3’s benchmark accuracy, particularly its accuracy in placing modified-nucleotide functional groups, is attributable to structures that were available during training. This pattern is most pronounced for modification-level accuracy rather than molecule type or modification type, reinforcing the interpretation above that fine-grained placement of nucleotide modifications is a comparatively weaker aspect of AlphaFold 3’s predictive capability than overall fold prediction.

## Discussion

This study provides a systematic classification of the chemical and structural landscape of modified nucleotides present in atomic structures deposited to the PDB (Fig. 1 and Supplementary Fig. S1). Hierarchical clustering of 1109 nucleotide CCD IDs based on physicochemical descriptors revealed that the landscape is shaped primarily by molecular size, topological shape, and polarity, rather than by modification type or biological origin, with chemically and functionally distinct modifications often clustering together based on shared molecular features. The majority of identified modified nucleotides (∼71%) were observed in structures in the PDB only as standalone ligands rather than as residues incorporated into DNA or RNA polymers. In addition, atomic structures of nucleic acids and nucleic acid–protein complexes containing modified nucleotides were heavily biased toward ~12 modified nucleotides (Supplementary Fig. S7). This result suggests that some types of modification in the chemical and structural space remain underexplored in nucleic acid polymers, since many standalone modifications appear structurally compatible with incorporation but have not yet been synthesized or resolved in that context. Future work synthesizing and solving structures of other types of modifications within DNA or RNA polymers, particularly those with bulky or topologically distinct substituents absent from current polymer structures (Fig. 1), could expand the known landscape of nucleic acid chemistry as well as inform nucleic acid therapeutic design.

We generated MODNAP, a curated dataset of 1039 experimentally determined structures spanning DNA, RNA, DNA–protein, RNA–protein, and DNA–RNA hybrid complexes representing 129 unique modified nucleotide CCD IDs (Fig. 2). MODNAP captures a partial portion of the chemical/structural space of nucleotide modifications present in the PDB, including base, sugar, and backbone modifications, and spans a wide range of biological functions, from natural epigenetic and regulatory modifications to synthetic molecules used in antisense, aptamer, and crystallographic phasing applications. As such, MODNAP represents a useful resource for benchmarking and, potentially, training next-generation structure prediction and co-folding algorithms on modified nucleic acids and nucleic acid–protein complexes. This area has received comparatively little attention relative to canonical, unmodified nucleic acid modeling [43, 55, 90, 128]. Using MODNAP, we performed a systematic benchmark of AlphaFold 3, the current state-of-the-art structure prediction and co-folding tool capable of modeling modified nucleotides via user-specified CCD identifiers [49]. AlphaFold 3 achieved high success rates across nearly all structural categories under standard RMSD, TM-score, and lDDT thresholds, and frequently placed modified functional groups in accurate register with the surrounding nucleic acid (Figs 4A–H and 5–9). However, a PoseBusters test suite analysis revealed that a substantial fraction of AlphaFold 3 models, despite close global agreement with experimental structures, contain intramolecular geometric violations, most notably non-ideal ribose ring puckering (Fig. 3A–C) [78]. Likewise, an RNAspider analysis supports the presence of entanglements in complex RNA folds (Supplementary Fig. S12). These discrepancies between RMSD-based accuracy and intramolecular chemical validity underscores an important caveat for the field: standard global superposition metrics alone are insufficient to assess the chemical and physical reliability of predicted modified nucleotide structures, and complementary validity checks should be considered standard practice when evaluating co-folding predictions intended for downstream mechanistic or therapeutic applications. Possible solutions to the problem include filtering out PoseBusters invalid or entangled AlphaFold 3 models and/or performing energy minimization to further relax the structure, fix bond lengths, and eliminate internal clashes [78].

This study has several limitations. First, we restricted our benchmarking analysis to AlphaFold 3. Other co-folding and structure prediction tools capable of modeling modified nucleotides, such as RoseTTAFold All-Atom [52], Chai-1 [51], and others, were not evaluated. Future studies should apply MODNAP to benchmark these and other modeling approaches, both to establish strengths and weaknesses across different methods and to identify whether alternative architectures handle ribose ring geometry, chemical validity, or generalization to unseen modifications more effectively. Second, although MODNAP was designed to provide broad representation of the modified nucleotide landscape, the dataset is unavoidably skewed toward a small number of well-studied modifications; roughly half of all CCD IDs in MODNAP are represented by only a single PDB structure (Supplementary Fig. S7). This imbalance reflects the underlying distribution of structures deposited in the PDB rather than a curation choice, but it limits the statistical power with which performance can be evaluated for rare or chemically unusual modifications and may bias aggregate performance metrics toward the modifications that are best represented. Relatedly, our benchmark stratified structures by whether they were deposited before or after AlphaFold 3’s training cutoff, and we observed a clear decline in both full-structure and modification-level accuracy for post-cutoff structures (Fig. 10C and D). While this comparison is informative, the post-cutoff subset (the MODNAP test set) is small (*n* = 103) and itself unevenly distributed across molecule types and modification types. Expanding MODNAP as new modified nucleic acid structures are deposited will improve the robustness of future generalization assessments and provide an evolving, versioned benchmark for tracking model improvement over time. Third, our curation pipeline applied several practical filters, including exclusion of NMR ensembles, resolution and sequence-length cutoffs, and removal of structures with unmodeled gaps, which were necessary to ensure high-confidence ground-truth coordinates and computational tractability but may have excluded structurally interesting or biologically important entries, such as larger ribonucleoprotein assemblies or more dynamic, sparsely populated nucleic acid conformations. Future expansions of MODNAP could incorporate nuclear magnetic resonance spectroscopy-derived ensembles, integrate dynamics-derived structural models, or relax sequence-length constraints as inference efficiency for larger complexes improves. Finally, we did not model non-ribose-based nucleic acids, such as 2′-deoxy-2′-fluoro-arabinonucleic acid, locked nucleic acids, hexitol nucleic acids, or acyclic nucleic acids, because these molecules are not represented in the MODNAP dataset. Although a limited number of experimentally determined structures of these types of nucleic acids are available in the Protein Data Bank, including structures containing CCD IDs XTR, 8XG, and 6HA, future studies should evaluate how AlphaFold 3 performs on these non-ribose-based nucleic acid systems.

## Supplementary Material

gkag999_Supplemental_File

## Data Availability

A list of modified nucleotide CCD IDs, the MODNAP benchmark files, associated scripts for running the benchmarks, and raw data used for analysis are provided at https://doi.org/10.5281/zenodo.22784529 and at GitHub at https://github.com/mcshanlab/Fu-et-al_MODNAP_2026.
