# Supplementary material for: The MODNAP benchmark set for modeling ribose-based modified nucleic acid and nucleic acid–protein complexes: a case study with AlphaFold 3
Source: Nucleic Acids Res. 2026 Oct 15;54(19):gkag999. doi: 10.1093/nar/gkag999 (PMC13669634; doi:10.1093/nar/gkag999)
Supplement: Supplementary file 1 [file gkag999_supplemental_file.pdf]

Supplementary Information for

The MODNAP Benchmark Set for Modeling Ribose-Based Modified Nucleic Acid and Nucleic Acid–Protein Complexes: A Case Study with AlphaFold 3

Jiemin Fu[1], Valeria Tohver Milam[2], Andrew C. McShan[1]*

[1]School of Chemistry and Biochemistry, Georgia Institute of Technology, Atlanta, GA 30332, USA

[2]School of Materials Science and Engineering, Georgia Institute of Technology, Atlanta, GA, USA

*Correspondence: Andrew C. McShan (andrew.mcshan@chemistry.gatech.edu)

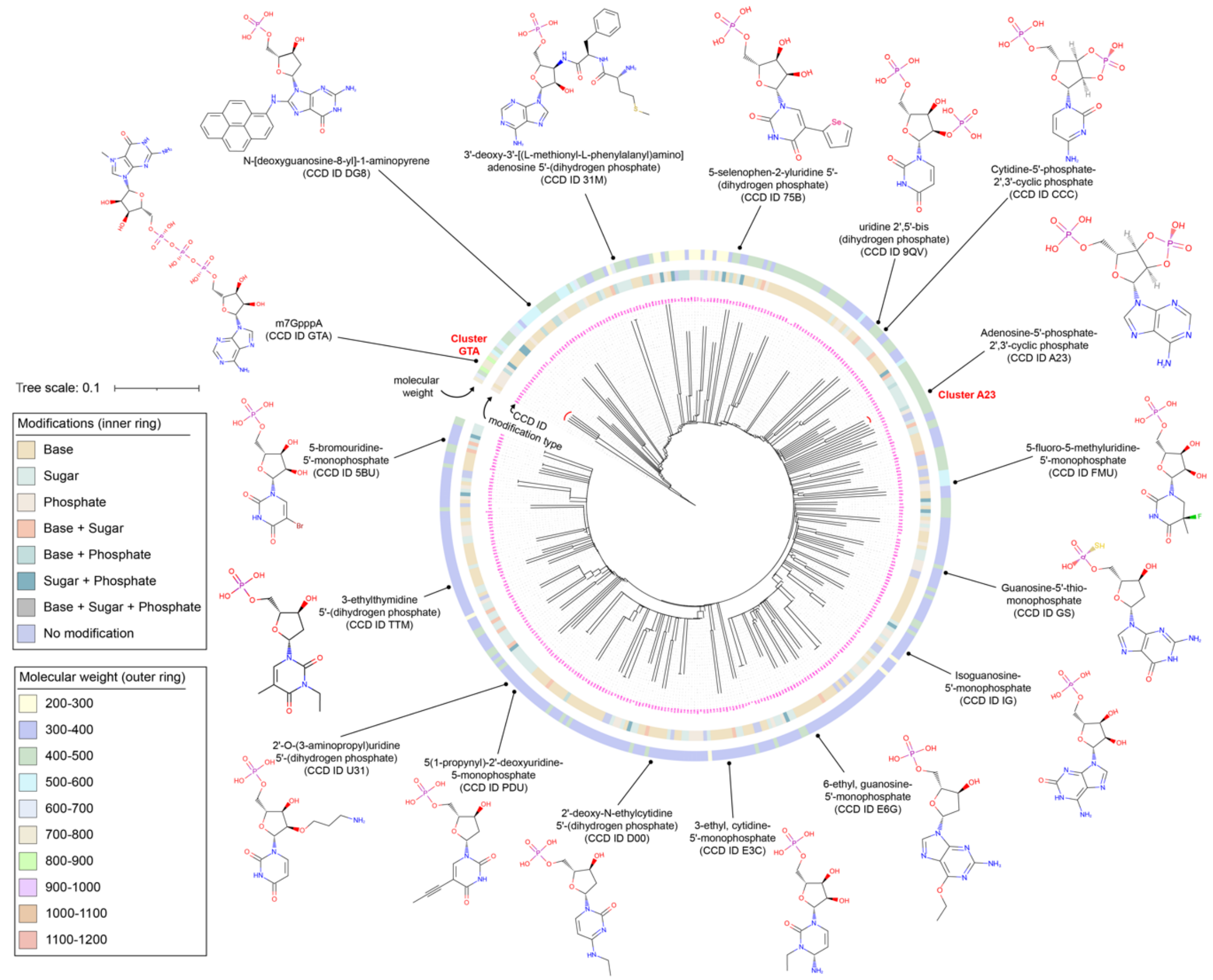

Supplementary Figure 1. Landscape of modified nucleotides present in nucleic acid polymers in the Protein Data Bank. A circular dendrogram of 375 modified nucleotides incorporated within DNA or RNA polymers in the PDB. A few unmodified natural nucleotides (A, G, T, C, U) were included for reference. The dendrogram was generated in iTOL v7 from a Newick tree derived from a ChemMine Tools analysis (SMILES → JOELib descriptors → hierarchical clustering → Newick tree). Chemical structures and CCD IDs of representative modified nucleotides are shown. The dendrogram inner ring is colored by modification type, while the outer ring is colored by molecular weight (Daltons, Da). The two red arches by the cluster branches denote clusters described in the main text (GTA cluster, top left; A23 cluster, top right).

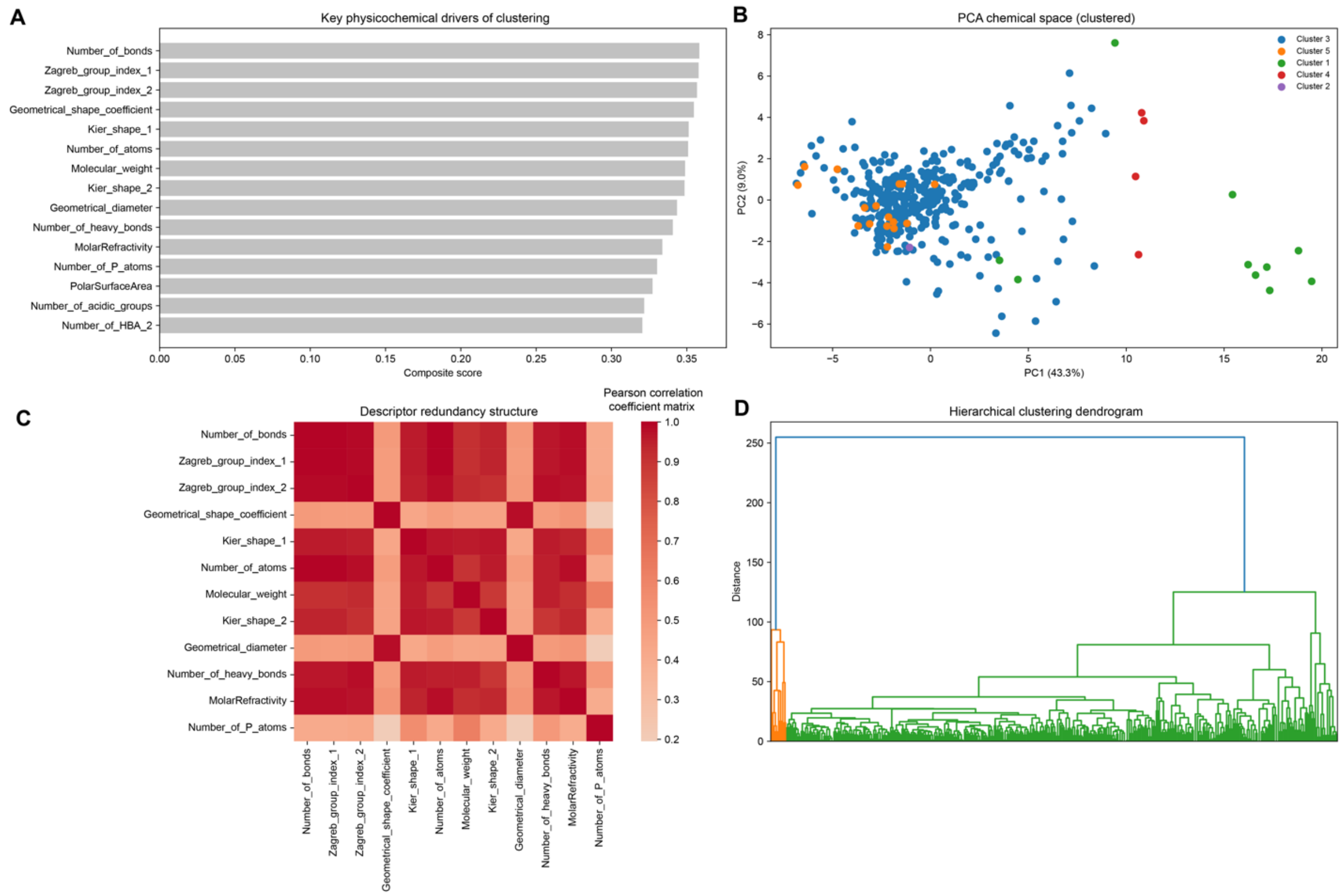

Supplementary Figure 2. JOELib physicochemical determinants of hierarchical clustering in modified nucleotides contained in DNA or RNA polymers in the PDB. (A) Ranked contributions of JOELib physicochemical descriptors to clustering structure, computed using a composite score integrating principal component analysis (PCA)-derived feature importance, mutual information with the first principal component (PC1), and redundancy penalties based on inter-feature correlations. (B) Projection of molecules into the first two principal components (PC1 and PC2) of the standardized descriptor space, with points colored according to hierarchical clustering assignment (k = 5). (C) Pearson correlation coefficient heatmap of the top contributing descriptors, visualizing pairwise relationships among features (red, positive correlation; blue, negative correlation). (D) Hierarchical clustering dendrogram generated from Euclidean distances of standardized descriptors using average linkage, with branch colors indicating cluster assignments defined by the chosen distance threshold.

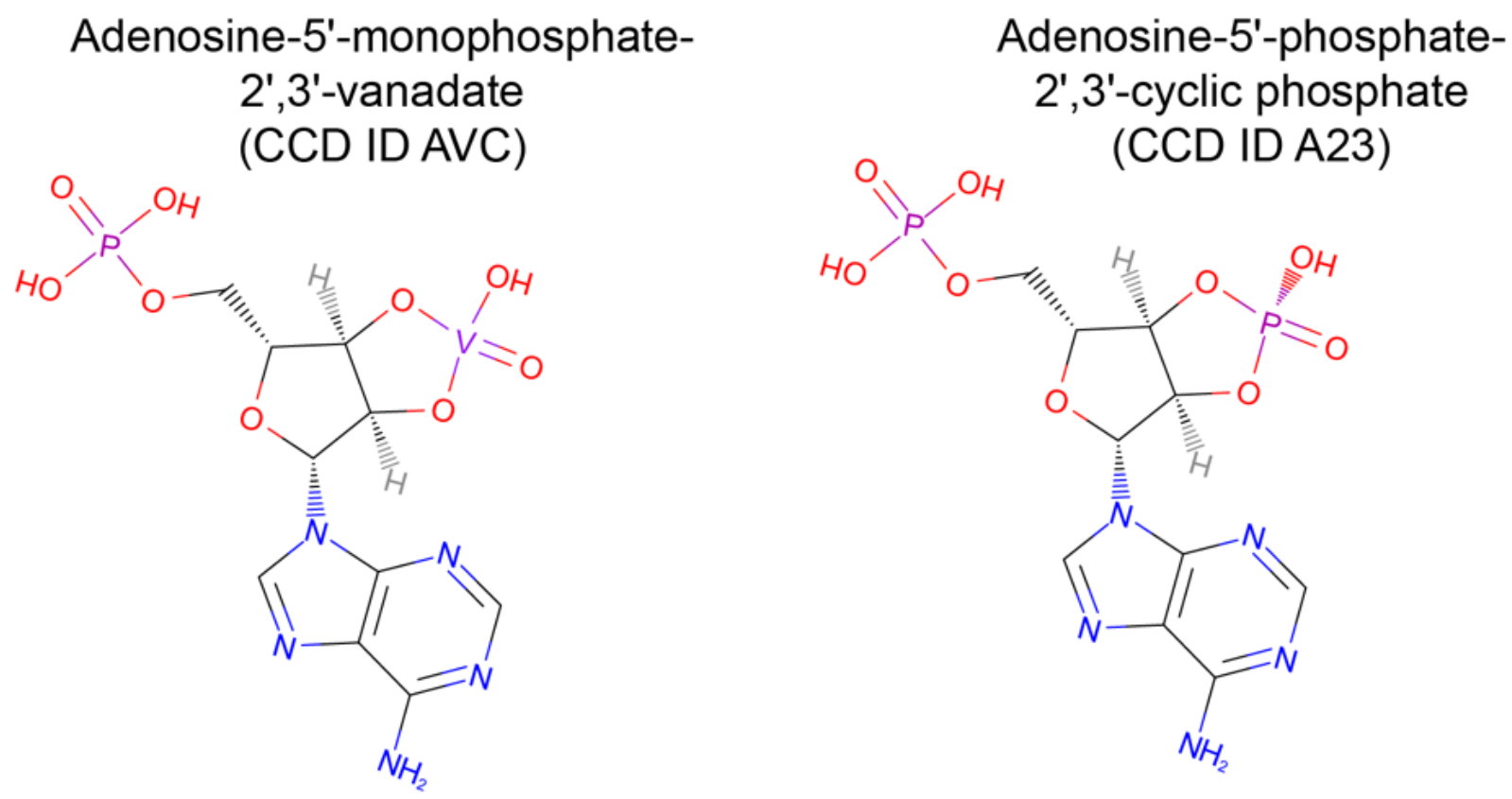

Supplementary Figure 3. Chemical structures and CCD IDs of modified nucleotides contained in the A23 cluster.

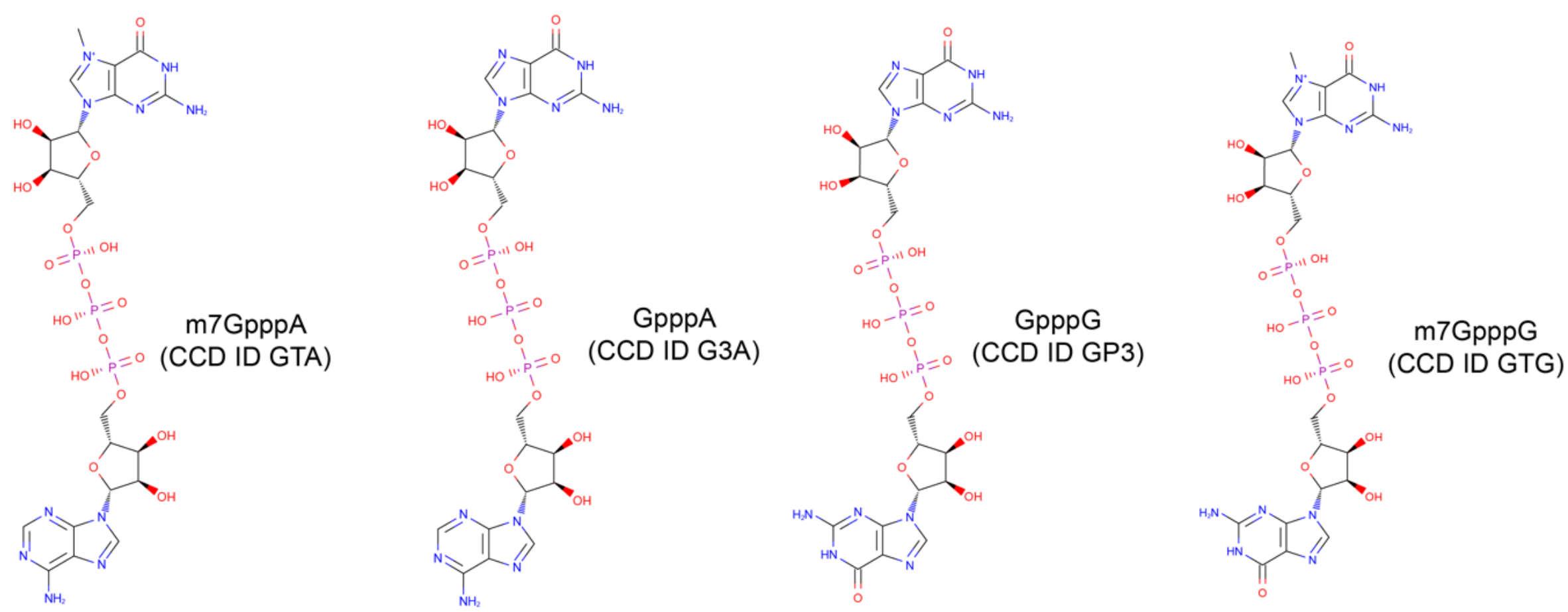

Supplementary Figure 4. Chemical structures and CCD IDs of modified nucleotides contained in the GTA cluster.

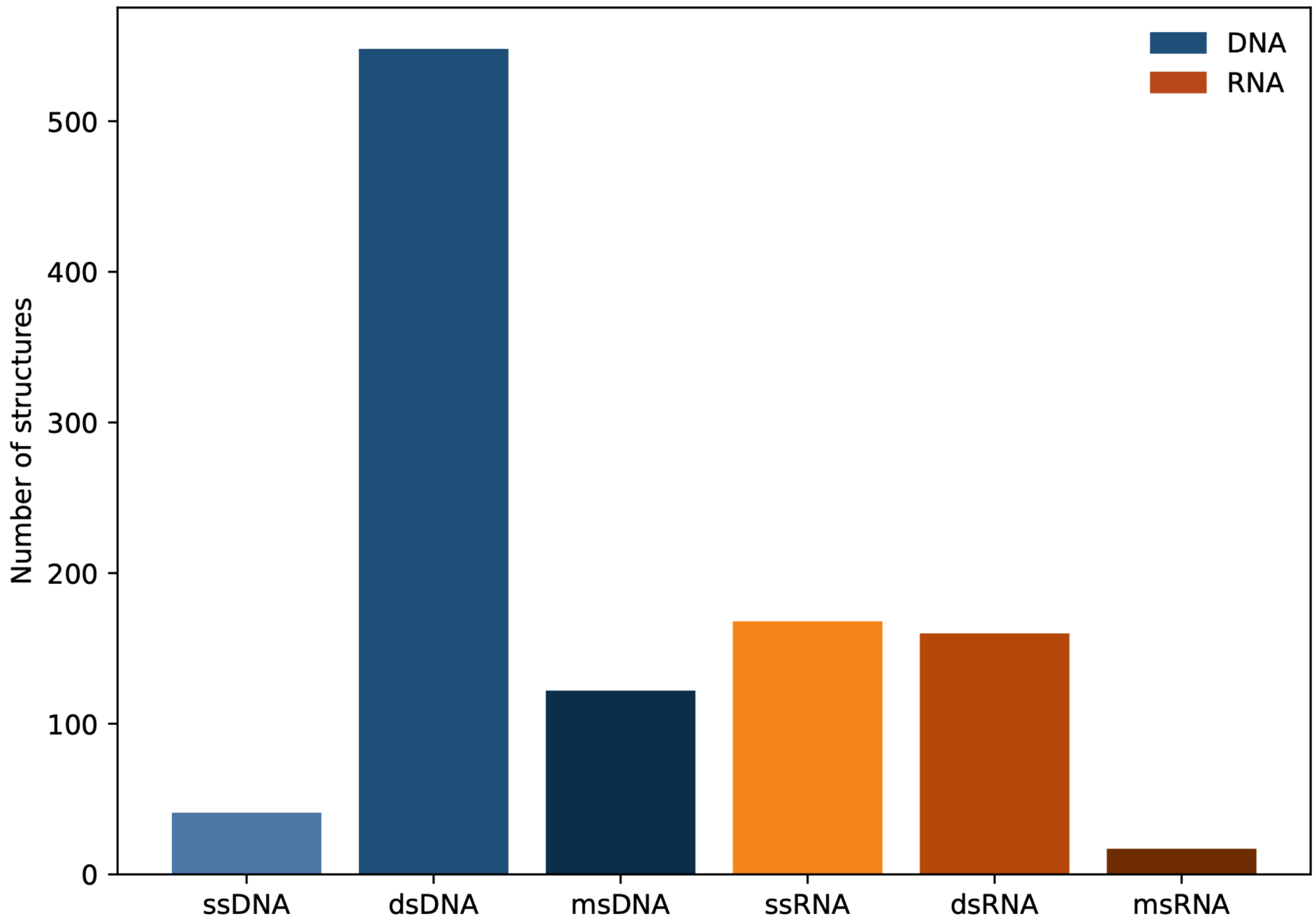

Supplementary Figure 5. Distribution of nucleic acid molecules in MODNAP entries. The number of structures containing single-stranded DNA (ssDNA), double-stranded DNA (dsDNA), multi-stranded DNA (msDNA), single-stranded RNA (ssRNA), double-stranded RNA (dsRNA), or multi-stranded RNA (msRNA) is shown. Multi-stranded refers to entries with more than two DNA or RNA strands.

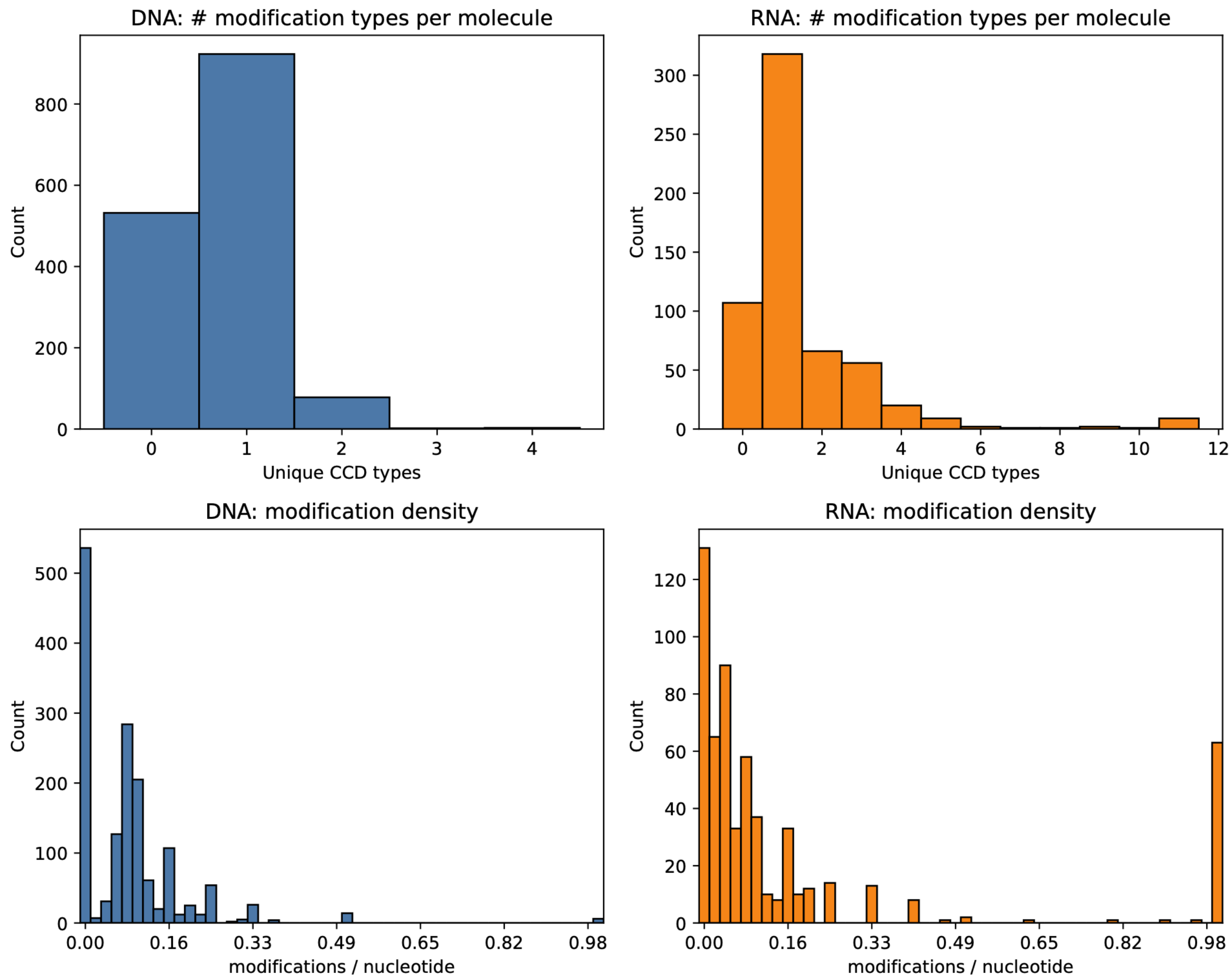

Supplementary Figure 6. Distribution of modified nucleotide features across MODNAP nucleic acid molecules. Histograms summarize modification patterns observed across DNA and RNA chains in the MODNAP dataset. The top panels show the distribution of the number of unique modification types (CCD identifiers) per DNA and RNA molecule. The bottom panels show the distribution of modification density, defined as the number of modification events normalized by sequence length (modifications per nucleotide). Each sequence chain is treated as an independent molecule-level observation. Some MODNAP entries contain at least one DNA or RNA sequence that is unmodified.

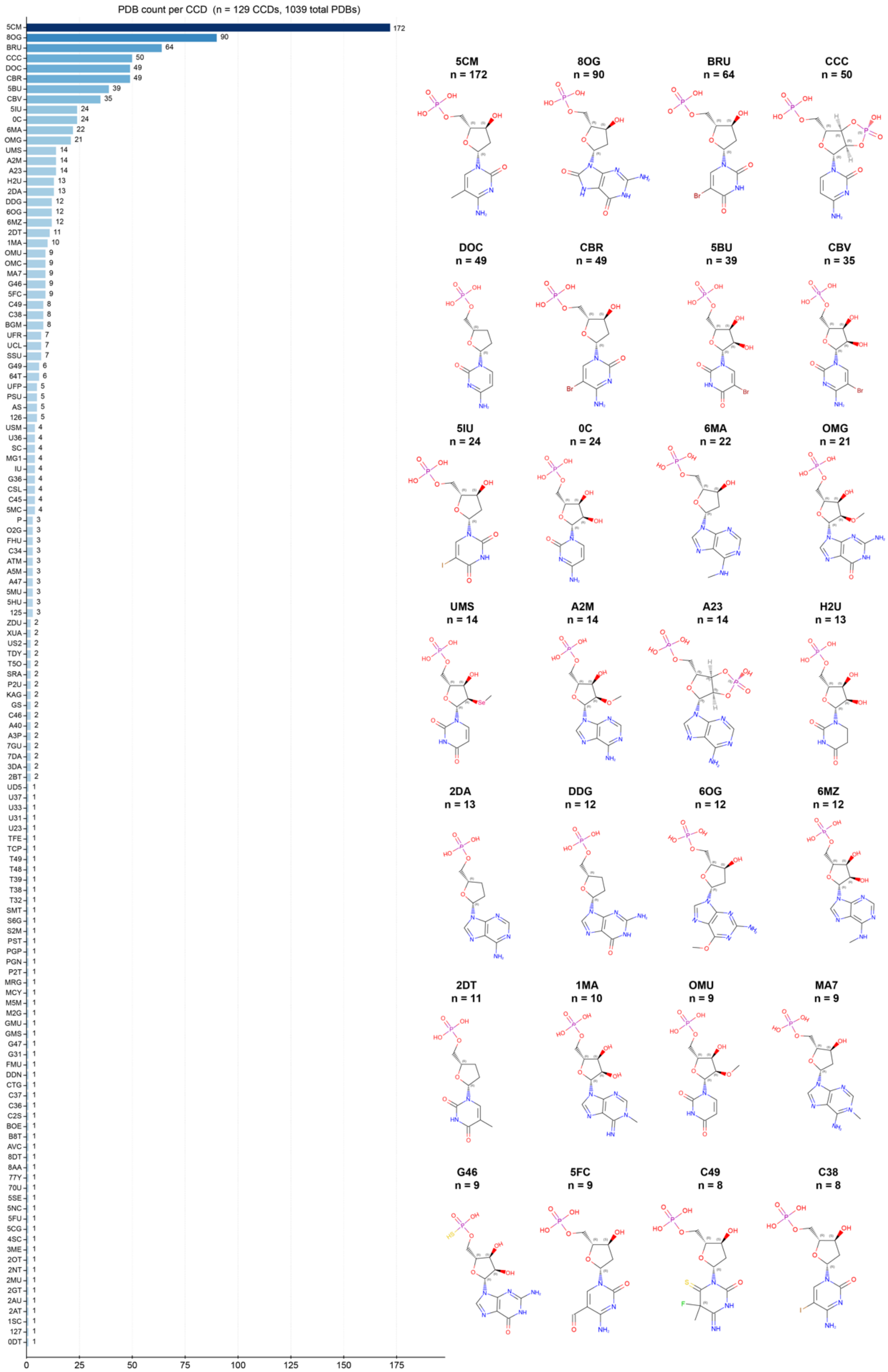

PDB count per CCD (n = 129 CCDs, 1039 total PDBs)
5CM 172
8OG 90
BRU 64
CCC 50
DOC 49
CBR 49
5BU 39
CBV 35
5IU 24
0C 24
6MA 22
OMG 21
UMS 14
A2M 14
A23 14
H2U 13
2DA 13
DDG 12
6OG 12
6MZ 12
2DT 11
1MA 10
OMU 9
OMC 9
MA7 9
G46 9
5FC 9
C49 8
C38 8
BGM 8
UFR 7
UCL 7
SSU 7
G49 6
64T 6
UFP 5
PSU 5
AS 5
126 5
USM 4
U36 4
SC 4
MG1 4
IU 4
G36 4
CSL 4
C45 4
5MC 4
P 3
O2G 3
FHU 3
C34 3
ATM 3
A5M 3
A47 3
5MU 3
5HU 3
125 3
ZDU 2
XUA 2
US2 2
TDY 2
T5O 2
SRA 2
P2U 2
KAG 2
GS 2
C46 2
A40 2
A3P 2
7GU 2
7DA 2
3DA 2
2BT 2
UD5 1
U37 1
U33 1
U31 1
U23 1
TFE 1
TCP 1
T49 1
T48 1
T39 1
T38 1
T32 1
SMT 1
S6G 1
S2M 1
PST 1
PGP 1
PGN 1
P2T 1
MRG 1
MCY 1
M5M 1
M2G 1
GMU 1
GMS 1
G47 1
G31 1
FMU 1
DDN 1
CTG 1
C37 1
C36 1
C2S 1
BOE 1
B8T 1
AVC 1
8DT 1
8AA 1
77Y 1
70U 1
5SE 1
5NC 1
5FU 1
5CG 1
4SC 1
3ME 1
2OT 1
2NT 1
2MU 1
2GT 1
2AU 1
2AT 1
1SC 1
127 1
0DT 1
0 25 50 75 100 125 150 175
Number of PDB structures
5CM n = 172
8OG n = 90
BRU n = 64
CCC n = 50
DOC n = 49
CBR n = 49
5BU n = 39
CBV n = 35
5IU n = 24
0C n = 24
6MA n = 22
OMG n = 21
UMS n = 14
A2M n = 14
A23 n = 14
H2U n = 13
2DA n = 13
DDG n = 12
6OG n = 12
6MZ n = 12
2DT n = 11
1MA n = 10
OMU n = 9
MA7 n = 9
G46 n = 9
5FC n = 9
C49 n = 8
C38 n = 8

Supplementary Figure 7. Distribution of PDB structures across modified nucleotide Chemical Component Dictionary (CCD) codes in MODNAP. Bar chart showing the number of PDB structures associated with each of the 129 modified nucleotide CCD codes present in MODNAP (1,039 total PDB structures). CCDs are ranked in descending order by structure count. Bar shading scales with count (darker blue = more structures). Chemical structures of CCD IDs abundant in MODNAP structures are shown.

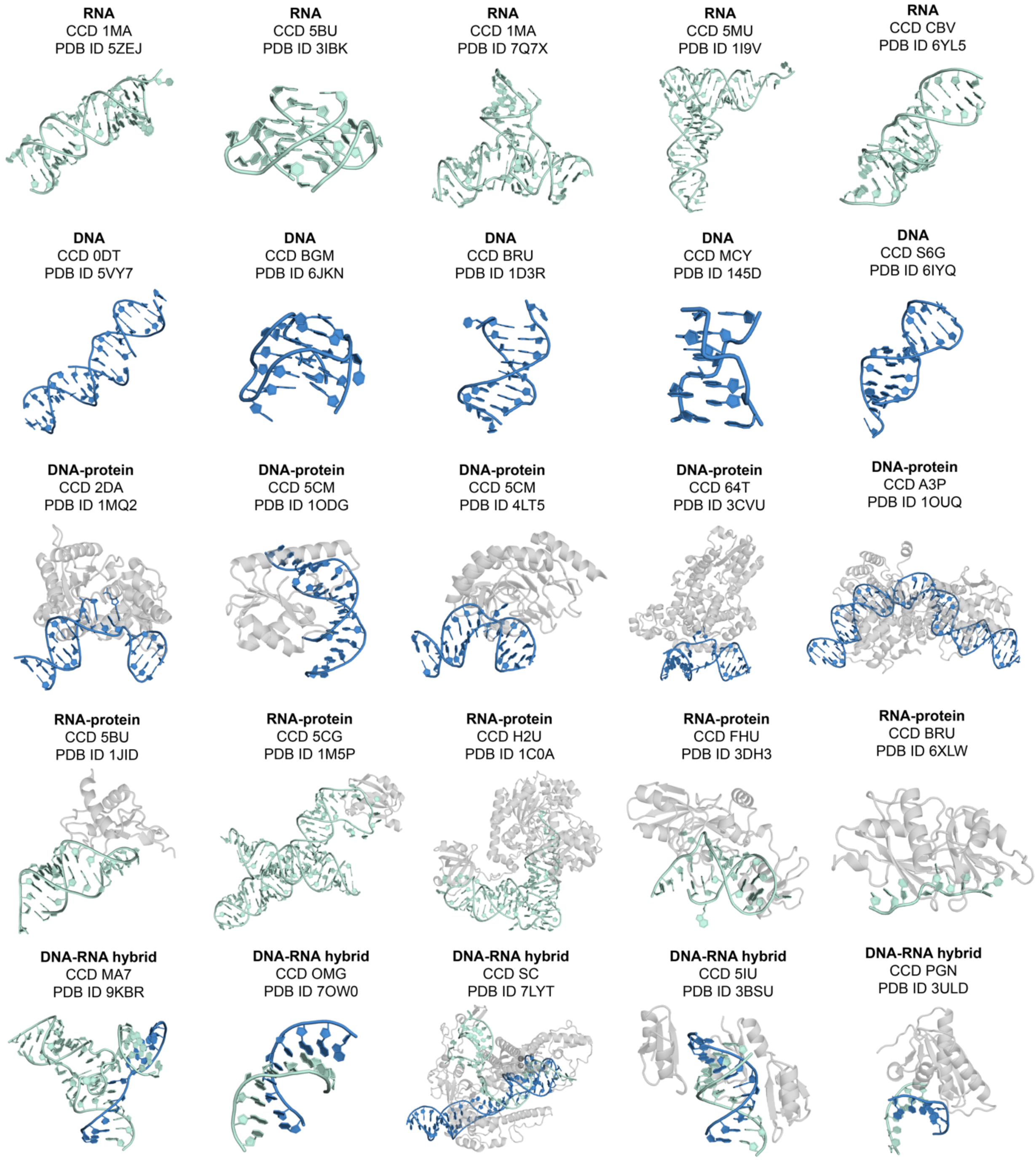

Supplementary Figure 8. Representative nucleic acid and nucleic acid–protein complex structures present in MODNAP. RNA, DNA, and protein molecules are colored green, blue, and gray, respectively. CCD IDs corresponding to the modified nucleotides and associated PDB IDs are provided.

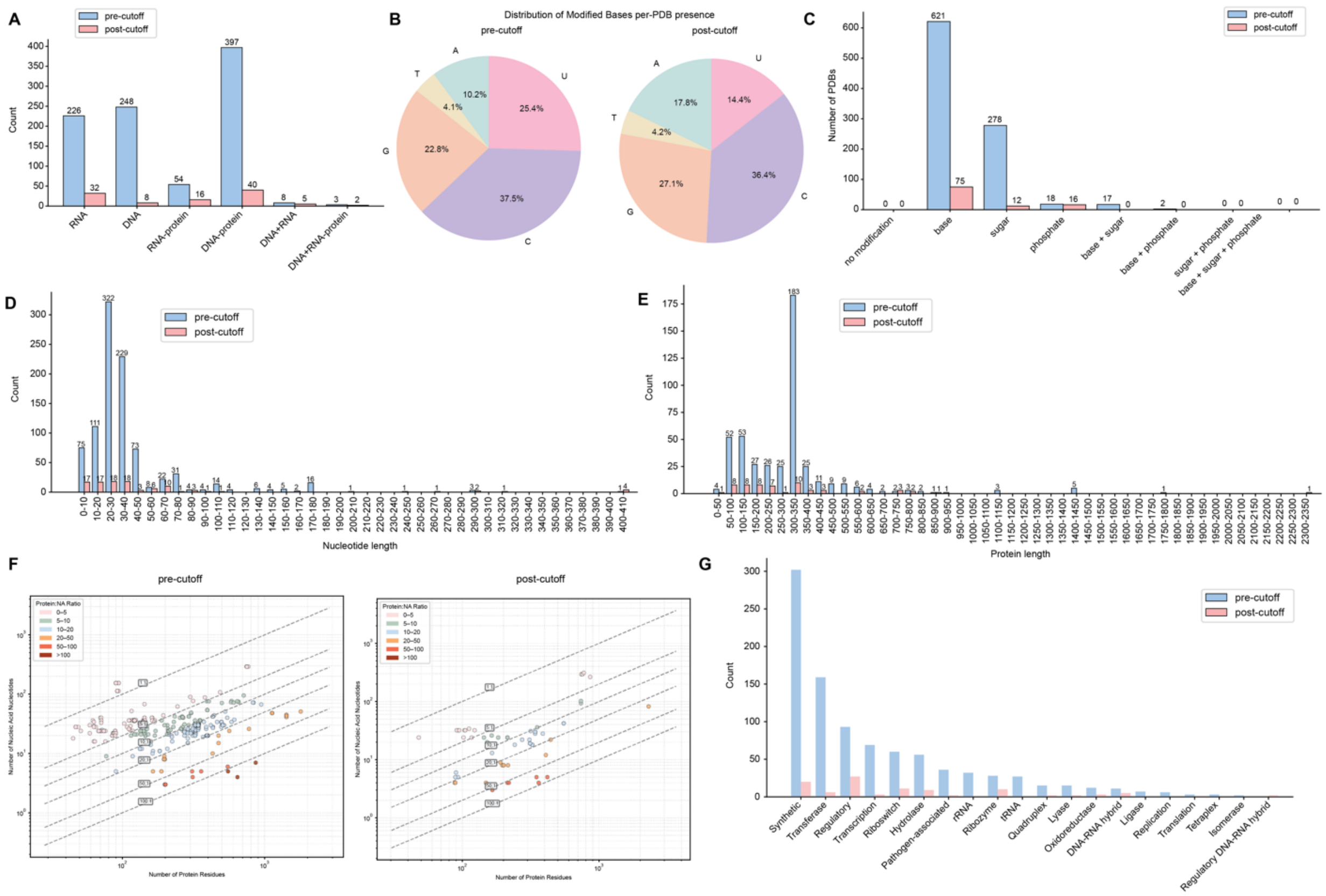

Supplementary Figure 9. Features of MODNAP test set (pre vs post cutoff statistics). The MODNAP test set (post-cutoff entries) represents 103 PDB structures released after AlphaFold 3's training cutoff date. **(A)** Total count of RNA, DNA, RNA–protein, DNA–protein, RNA/DNA hybrid, and DNA/RNA hybrid–protein structures in MODNAP for pre- vs post-cutoff entries. **(B)** Distribution of modified nucleobases (A, T, G, C, and U) by percentage in MODNAP for pre- vs post-cutoff entries. **(C)** Total number of PDB entries containing different modification types in MODNAP for pre- vs post-cutoff entries. **(D)** Total count of entries in MODNAP containing nucleic acid lengths in binned intervals of 10 nucleotides for pre- vs post-cutoff entries. **(E)** Total count of entries in MODNAP containing protein lengths in binned intervals of 50 amino acids for pre- vs post-cutoff entries. **(F)** Distribution of protein-to-nucleic acid length ratios (number of protein amino acids / number of nucleotides) across complexes in MODNAP for pre- vs post-cutoff entries. **(G)** Total count of annotated functions of nucleic acid or nucleic acid–protein complex in MODNAP for pre- vs post-cutoff entries. For nucleic acid–protein complex entries, the functional annotation of the protein is provided.

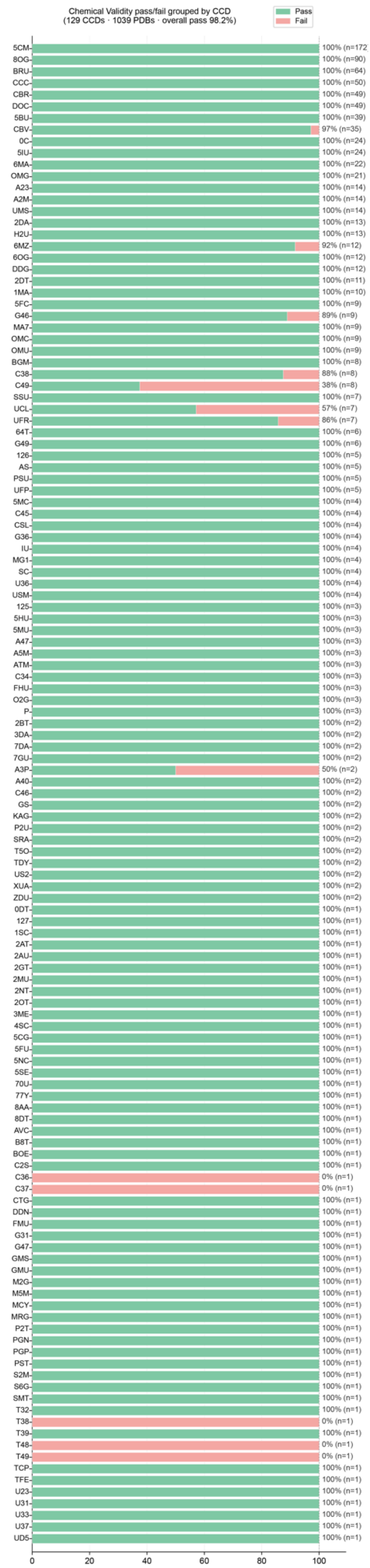

Chemical Validity pass/fail grouped by CCD
(129 CCDs · 1039 PDBs · overall pass 98.2%)
Pass
Fail
5CM 100% (n=172
8OG 100% (n=90)
BRU 100% (n=64)
CCC 100% (n=50)
CBR 100% (n=49)
DOC 100% (n=49)
5BU 100% (n=39)
CBV 97% (n=35)
0C 100% (n=24)
5IU 100% (n=24)
6MA 100% (n=22)
OMG 100% (n=21)
A23 100% (n=14)
A2M 100% (n=14)
UMS 100% (n=14)
2DA 100% (n=13)
H2U 100% (n=13)
6MZ 92% (n=12)
6OG 100% (n=12)
DDG 100% (n=12)
2DT 100% (n=11)
1MA 100% (n=10)
5FC 100% (n=9)
G46 89% (n=9)
MA7 100% (n=9)
OMC 100% (n=9)
OMU 100% (n=9)
BGM 100% (n=8)
C38 88% (n=8)
C49 38% (n=8)
SSU 100% (n=7)
UCL 57% (n=7)
UFR 86% (n=7)
64T 100% (n=6)
G49 100% (n=6)
126 100% (n=5)
AS 100% (n=5)
PSU 100% (n=5)
UFP 100% (n=5)
5MC 100% (n=4)
C45 100% (n=4)
CSL 100% (n=4)
G36 100% (n=4)
IU 100% (n=4)
MG1 100% (n=4)
SC 100% (n=4)
U36 100% (n=4)
USM 100% (n=4)
125 100% (n=3)
5HU 100% (n=3)
5MU 100% (n=3)
A47 100% (n=3)
A5M 100% (n=3)
ATM 100% (n=3)
C34 100% (n=3)
FHU 100% (n=3)
O2G 100% (n=3)
P 100% (n=3)
2BT 100% (n=2)
3DA 100% (n=2)
7DA 100% (n=2)
7GU 100% (n=2)
A3P 50% (n=2)
A40 100% (n=2)
C46 100% (n=2)
GS 100% (n=2)
KAG 100% (n=2)
P2U 100% (n=2)
SRA 100% (n=2)
T5O 100% (n=2)
TDY 100% (n=2)
US2 100% (n=2)
XUA 100% (n=2)
ZDU 100% (n=2)
0DT 100% (n=1)
127 100% (n=1)
1SC 100% (n=1)
2AT 100% (n=1)
2AU 100% (n=1)
2GT 100% (n=1)
2MU 100% (n=1)
2NT 100% (n=1)
2OT 100% (n=1)
3ME 100% (n=1)
4SC 100% (n=1)
5CG 100% (n=1)
5FU 100% (n=1)
5NC 100% (n=1)
5SE 100% (n=1)
70U 100% (n=1)
77Y 100% (n=1)
8AA 100% (n=1)
8DT 100% (n=1)
AVC 100% (n=1)
B8T 100% (n=1)
BOE 100% (n=1)
C2S 100% (n=1)
C36 0% (n=1)
C37 0% (n=1)
CTG 100% (n=1)
DDN 100% (n=1)
FMU 100% (n=1)
G31 100% (n=1)
G47 100% (n=1)
GMS 100% (n=1)
GMU 100% (n=1)
M2G 100% (n=1)
M5M 100% (n=1)
MCY 100% (n=1)
MRG 100% (n=1)
P2T 100% (n=1)
PGN 100% (n=1)
PGP 100% (n=1)
PST 100% (n=1)
S2M 100% (n=1)
S6G 100% (n=1)
SMT 100% (n=1)
T32 100% (n=1)
T38 0% (n=1)
T39 100% (n=1)
T48 0% (n=1)
T49 0% (n=1)
TCP 100% (n=1)
TFE 100% (n=1)
U23 100% (n=1)
U31 100% (n=1)
U33 100% (n=1)
U37 100% (n=1)
UD5 100% (n=1)
0
20
40
60
80
100
Percentage of AlphaFold 3 models within each CCD (%)

Supplementary Figure 10. Distribution of AlphaFold 3 models of MODNAP entries by CCD ID that pass (green) or fail (red) PoseBuster's chemical validity test. Percentages indicate the fraction of models that pass. n denotes the number of MODNAP entries modeled for each CCD ID.

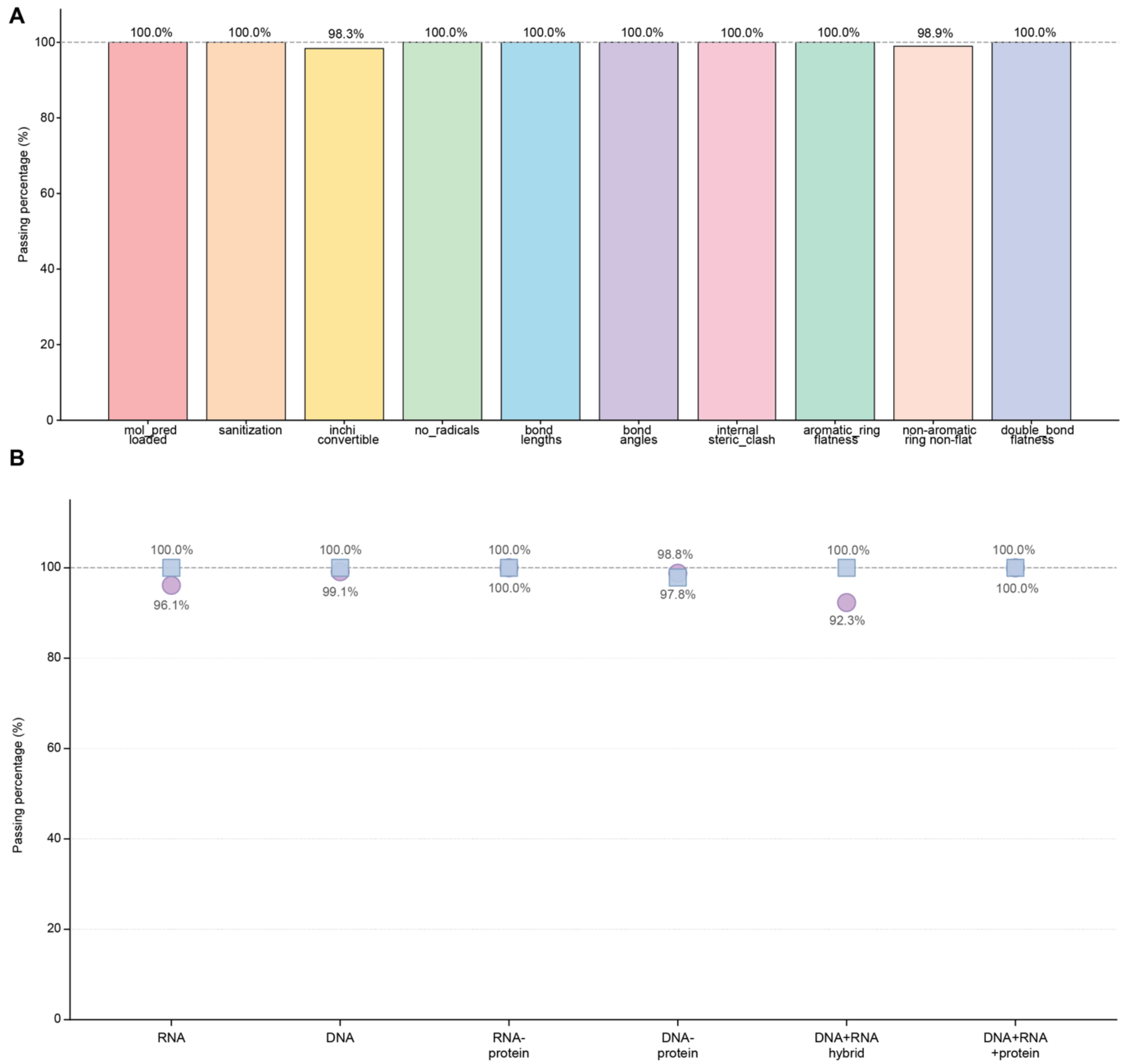

Supplementary Figure 11. PoseBusters test suite validity of natural unmodified nucleotides in AlphaFold 3 generated models of modified nucleic acids and nucleic acid–protein complexes. (A) Passing percentage of natural unmodified nucleotides in AlphaFold 3 models of all MODNAP entries as a function of each PoseBusters test-suite descriptors of chemical validity and intramolecular validity. (B) Passing percentage of chemical validity and intramolecular validity terms for natural unmodified nucleotides in AlphaFold 3 models of all MODNAP entries as a function of molecule type.

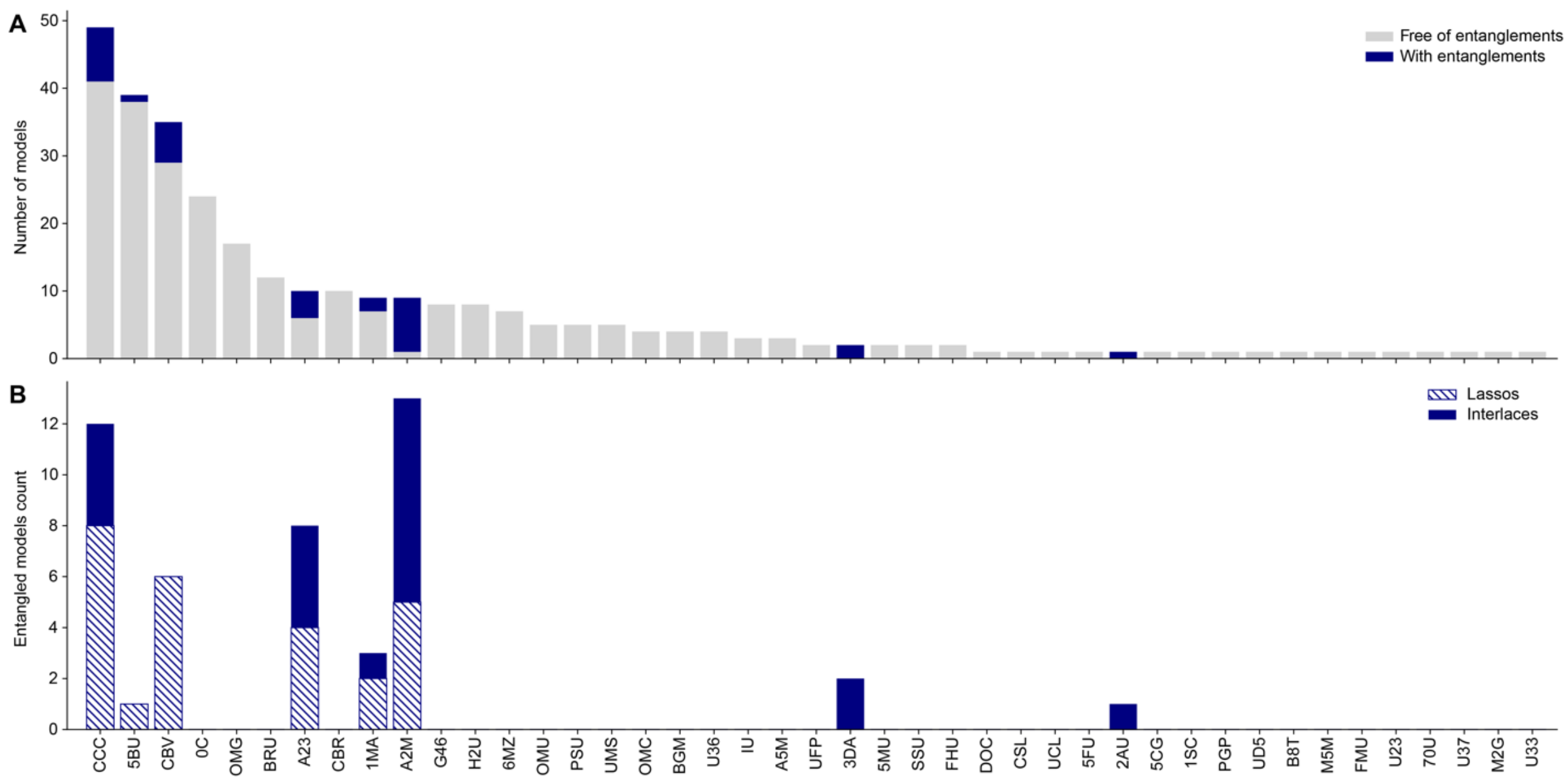

Supplementary Figure 12. Assessments of entanglements in AlphaFold 3 models of MODNAP entries using RNAspider (80). **(A)** Number of MODNAP entries for which AlphaFold 3 predicted models with or without entanglements, grouped by CCD ID. **(B)** Distribution of lasso- and interlace-type entanglements identified in the AlphaFold 3 models.

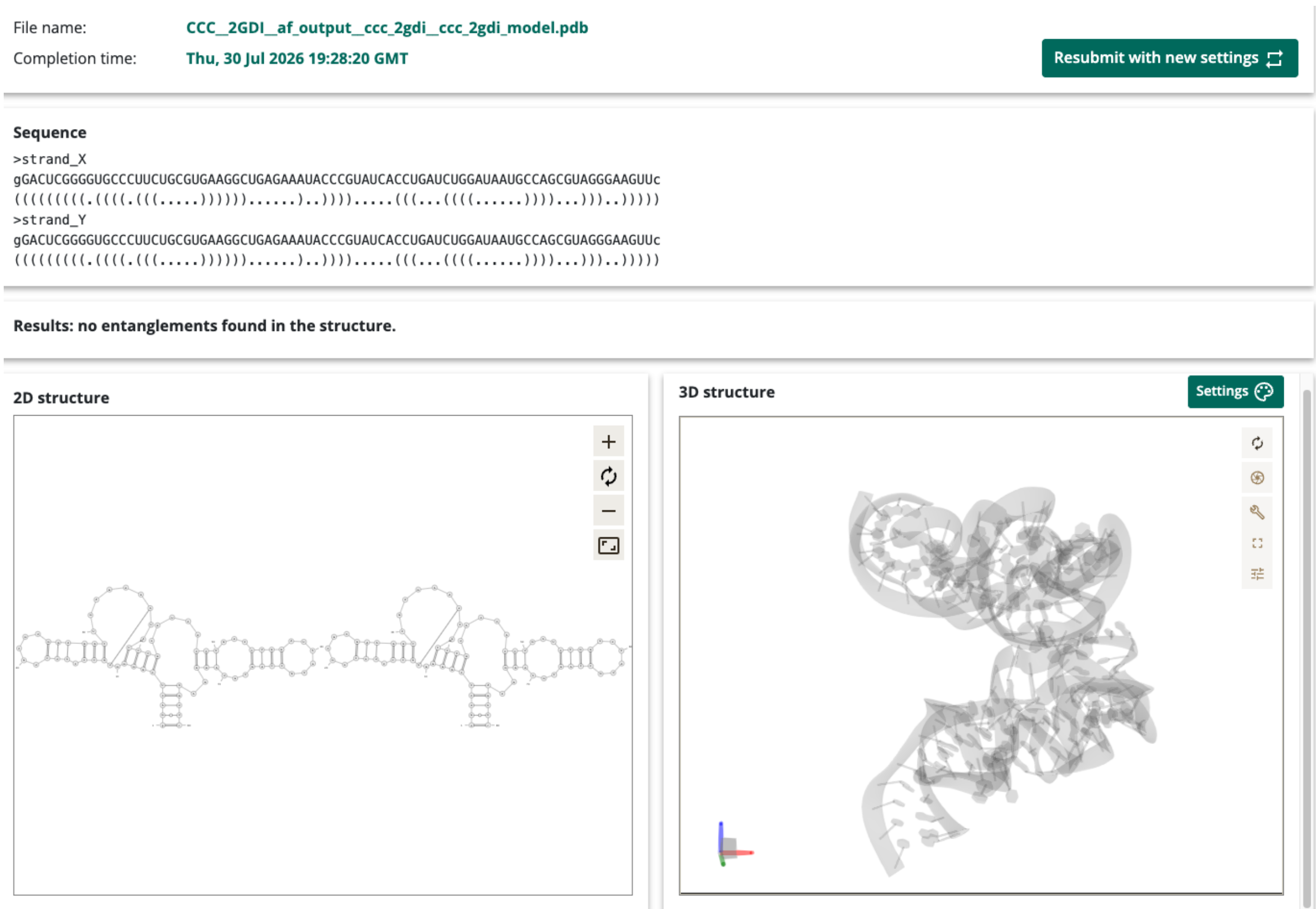

Supplementary Figure 13. Snapshot of the RNAspider webserver results for an AlphaFold 3 model of MODNAP entry PDB ID 2GDI (CCD ID CCC) showing no detected entanglements (80).

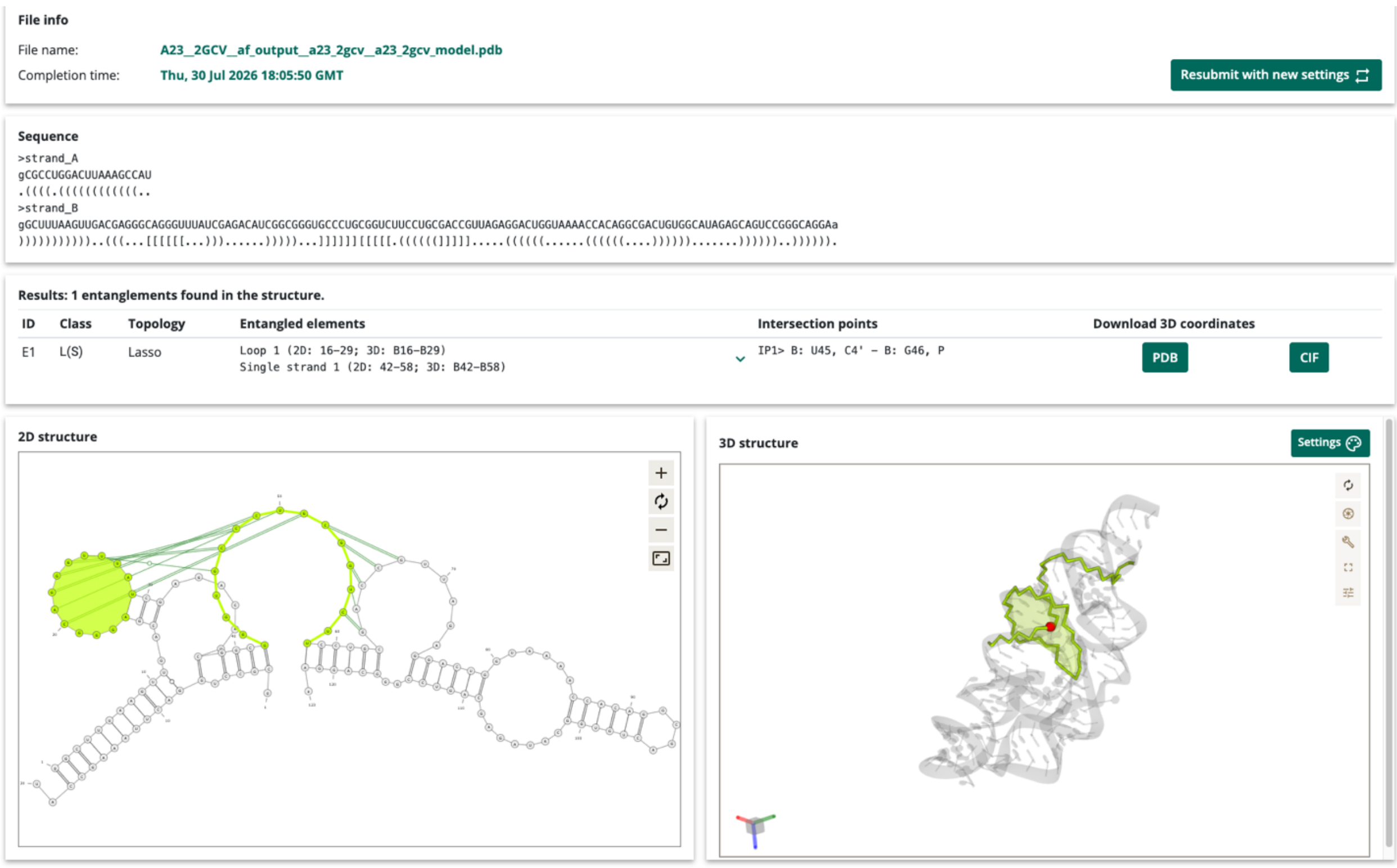

Supplementary Figure 13 continued. Snapshot of the RNAspider webserver results for an AlphaFold 3 model of MODNAP entry PDB ID 2GCV (CCD ID A23) showing a lasso-type entanglement (80).

File name: A2M_3L3C_af_output_a2m_3l3c_a2m_3l3c_model.pdb
Completion time: Thu, 30 Jul 2026 17:39:11 GMT

Resubmit with new settings

Sequence

```
>strand_E
aaGCGCCAGAACU
...((((.(((((
>strand_P
gGCACCAUUGCACUCCGGUGCCAGUUGACGAGGUGGGGUUUAUCGAGAUUUCGGCGGAUGACUCCCGGUUGUUCAUCACAACCGCAAGCUUUUACUUAAAUCAUUAAGGUGACUUAGUGGACAAAGGUGAAAGUGUGAUGA
((((((..........))))))))))..(((...[[[[[[..)))......)))))...]]]]]](((((((..[[[[[)))))))...(((((((((.....(((((((....))))))).......))))))))).]]]]].
```

Results: 1 entanglements found in the structure.

ID	Class	Topology	Entangled elements	Intersection points	Download 3D coordinates	
E1	L(S)	Lasso	Loop 1 (2D: 31–43; 3D: P31–P43)<br>Single strand 1 (2D: 56–66; 3D: P56–P66)	IP1> P: U59, C4' – P: G60, P	PDB	CIF

2D structure

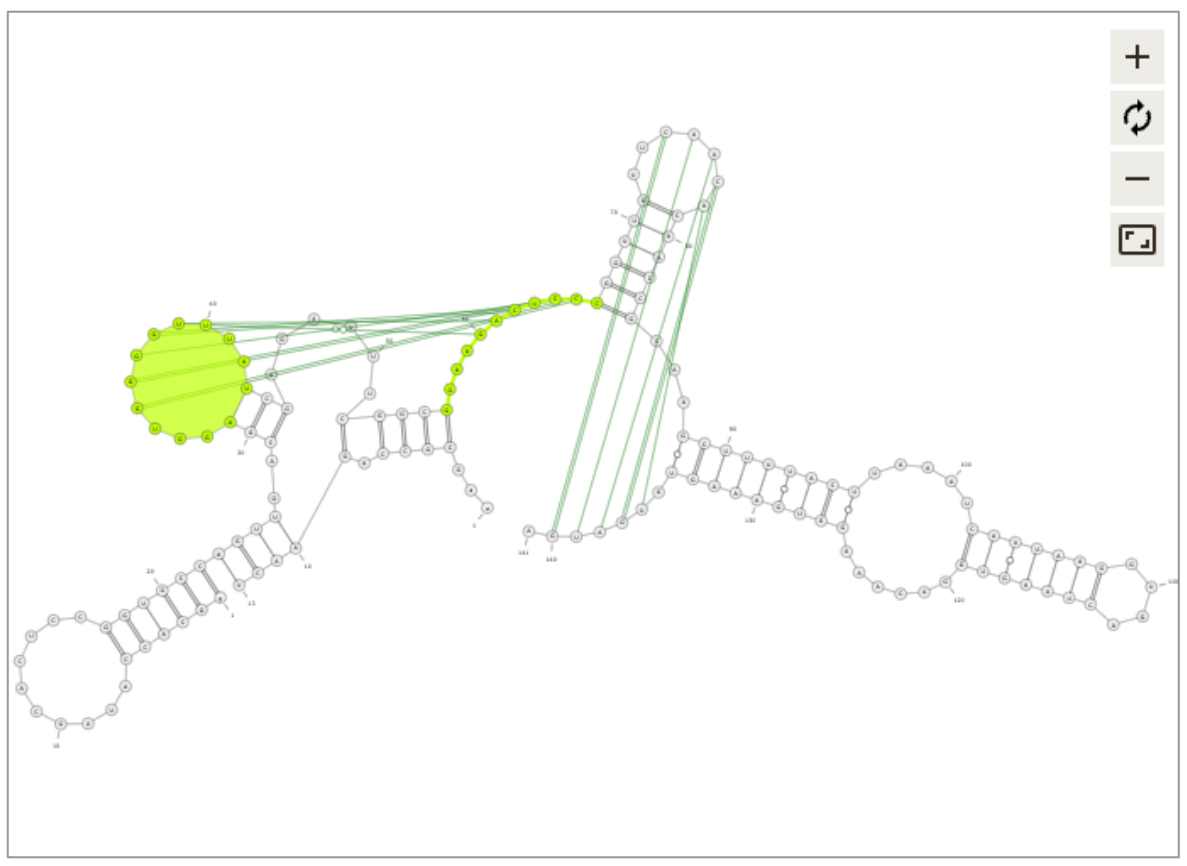

3D structure Settings

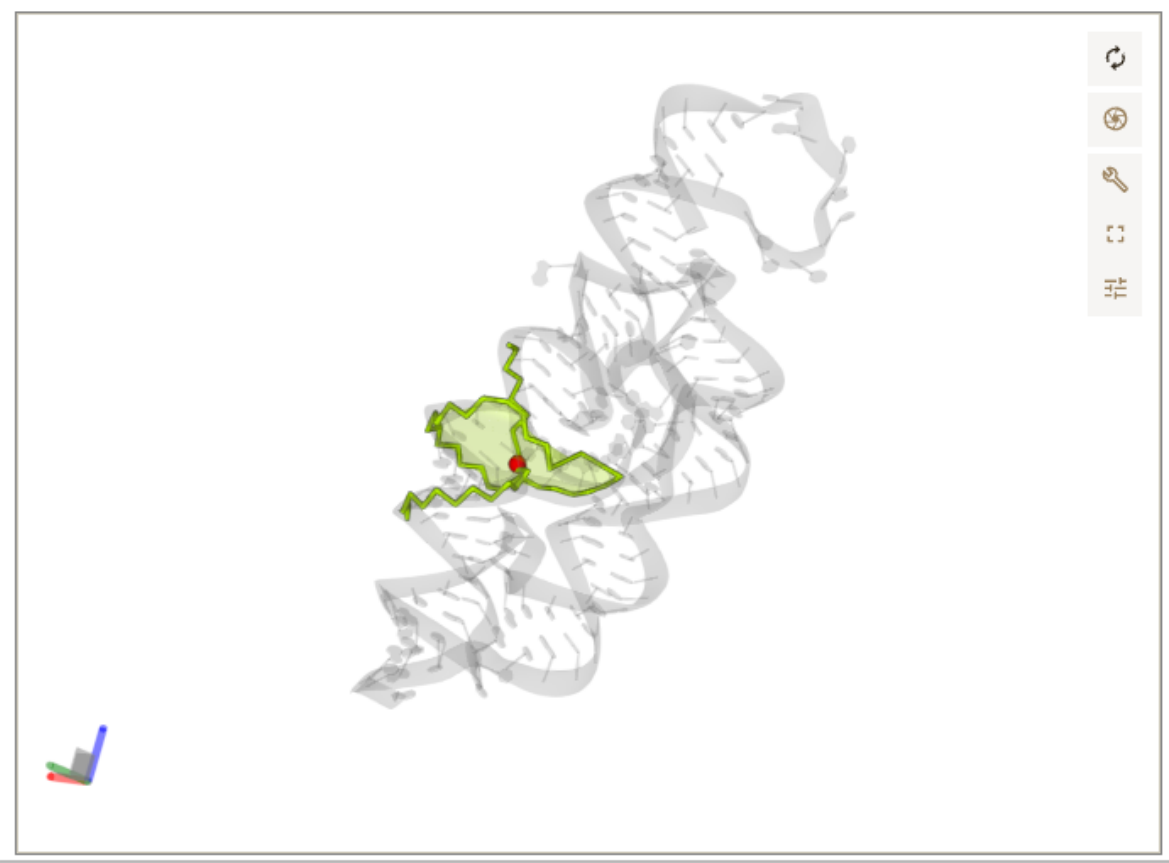

Supplementary Figure 13 continued. Snapshot of the RNAspider webserver results for an AlphaFold 3 model of MODNAP entry PDB ID 3L3C (CCD ID A2M) showing a lasso-type entanglement (80).

CCD ID	PDB ID	ENTANGLEMENT TYPE	MOLECULE TYPE
1MA	7Q7X	Lasso	Ribozyme
1MA	7Q82	Lasso, Interlace	Ribozyme
2AU	2NPY	Interlace	Ribozyme
3DA	2P7F	Interlace	Ribozyme
3DA	4G6R	Interlace	Ribozyme
5BU	2JLT	Lasso	Pathogen-associated
A23	2GCV	Lasso, Interlace	Ribozyme
A23	2H0W	Lasso, Interlace	Ribozyme
A23	2HO6	Lasso, Interlace	Ribozyme
A23	2HO7	Lasso, Interlace	Ribozyme
A2M	1X9C	Interlace	Ribozyme
A2M	1ZFT	Interlace	Ribozyme
A2M	2D2K	Interlace	Ribozyme
A2M	2NZ4	Lasso, Interlace	Ribozyme
A2M	3G8S	Lasso, Interlace	Ribozyme
A2M	3G8T	Lasso, Interlace	Ribozyme
A2M	3G96	Lasso, Interlace	Ribozyme
A2M	3L3C	Lasso, Interlace	Ribozyme
CBV	6R47	Lasso	Ribozyme
CBV	6YL5	Lasso	Riboswitch
CBV	6YLB	Lasso	Riboswitch
CBV	6YMK	Lasso	Riboswitch
CBV	6YML	Lasso	Riboswitch
CBV	6YMM	Lasso	Riboswitch
CCC	2QUW	Lasso, Interlace	Ribozyme
CCC	6DLQ	Lasso	Riboswitch
CCC	6DLR	Lasso	Riboswitch
CCC	6DLS	Lasso	Riboswitch
CCC	6DLT	Lasso	Riboswitch
CCC	9LKU	Lasso, Interlace	Riboswitch
CCC	9LKV	Lasso, Interlace	Riboswitch
CCC	9UW0	Lasso, Interlace	Riboswitch

Supplementary Figure 14. List of MODNAP entries for which AlphaFold 3 models were identified by RNAspider as containing entanglements, including the corresponding PDB IDs, CCD IDs, and molecule types. Most entries correspond to ribozyme or riboswitch folds.

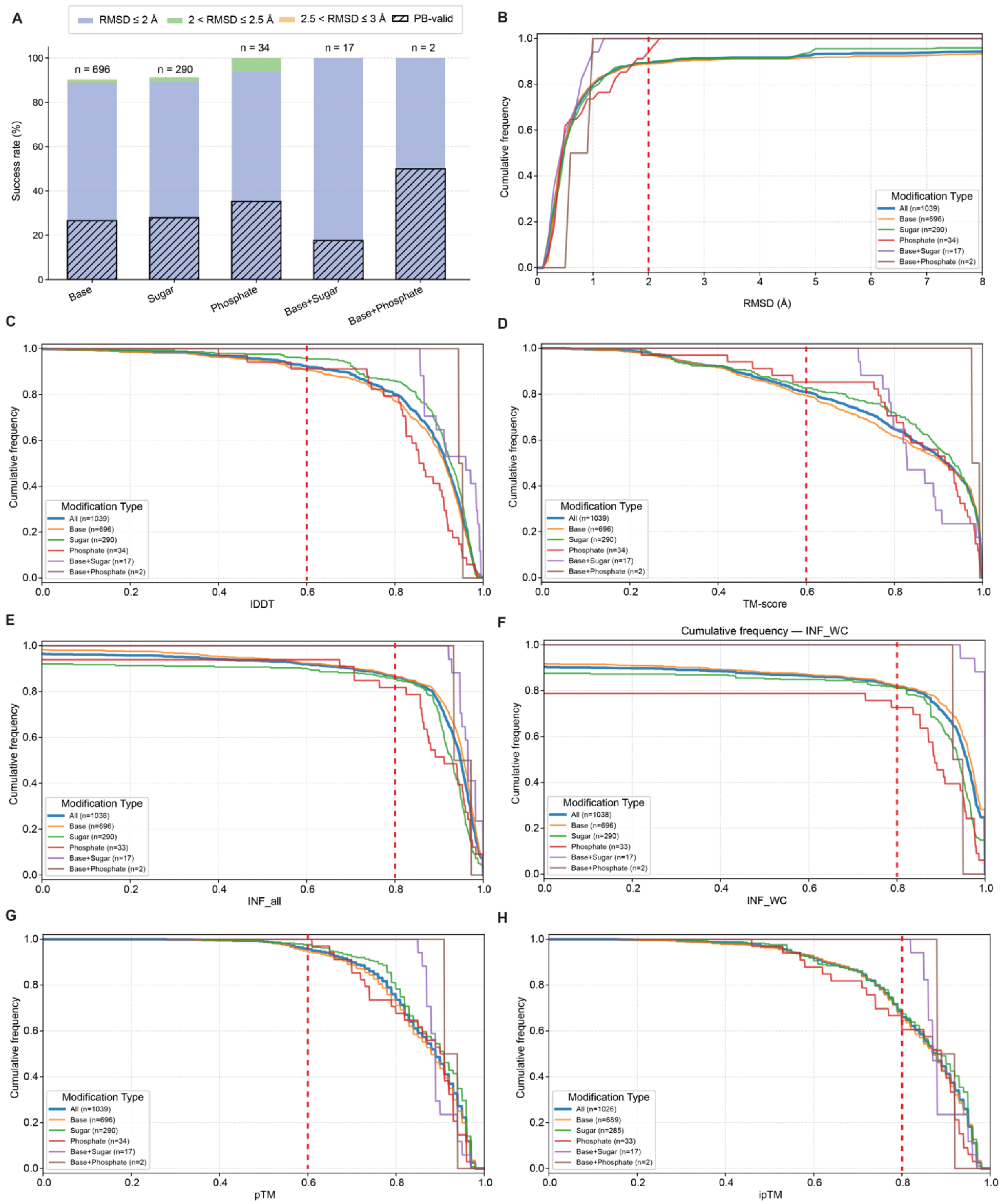

Supplementary Figure 15. Performance of AlphaFold 3 on the MODNAP benchmark set by modification type. (A) Bar plot of the success rates under RMSD threshold values of 2 Å, 2.5 Å, or 3 Å, and PoseBusters valid (PB-valid). Success rates for RMSD thresholds are defined as the percentages of predictions under the specific RMSD threshold values. Success rates for PB-valid cutoff are defined as the percentages of predictions that passed each chemical validity and

intramolecular validity tests and were labeled in dark shades. Cumulative frequency for **(B)** RMSD with a threshold value of 8 Å, **(C)** lDDT with a threshold value of 1, **(D)** TM-score with a threshold value of 1, **(E)** INF_all values with a maximum value of 1, **(F)** INF_WC values with a maximum value of 1, **(G)** pTM values with a threshold value of 1, and **(H)** ipTM values with a threshold value of 1. The dotted red lines represent standard threshold values for modeling accuracy.

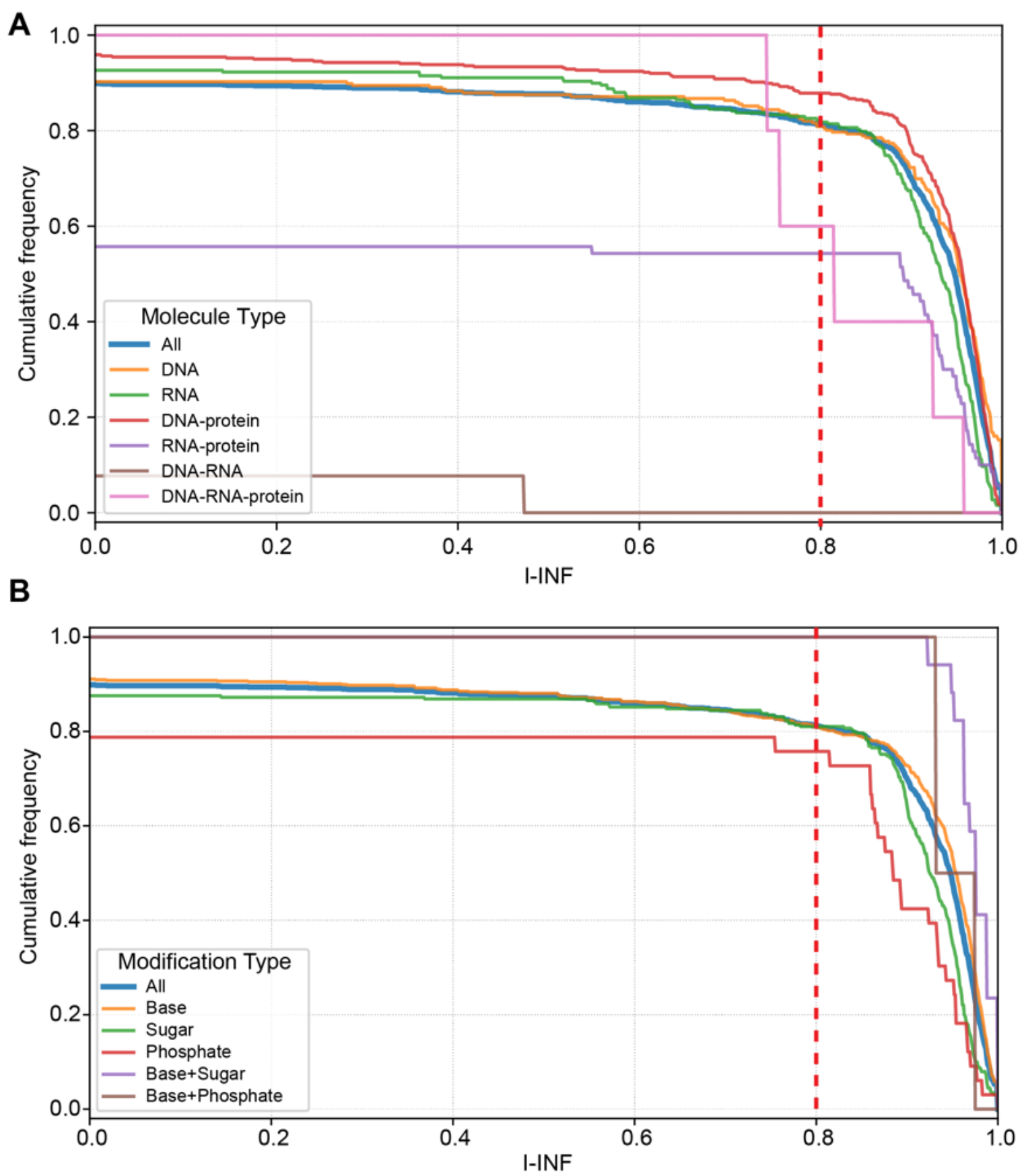

Supplementary Figure 16. Cumulative frequency for Interaction INF (I-INF) values calculated for AlphaFold 3 models of MODNAP entries relative to experimentally determined structures categorized by **(A)** molecule type, and **(B)** modification type. The dotted red lines represent standard threshold values for modeling accuracy.

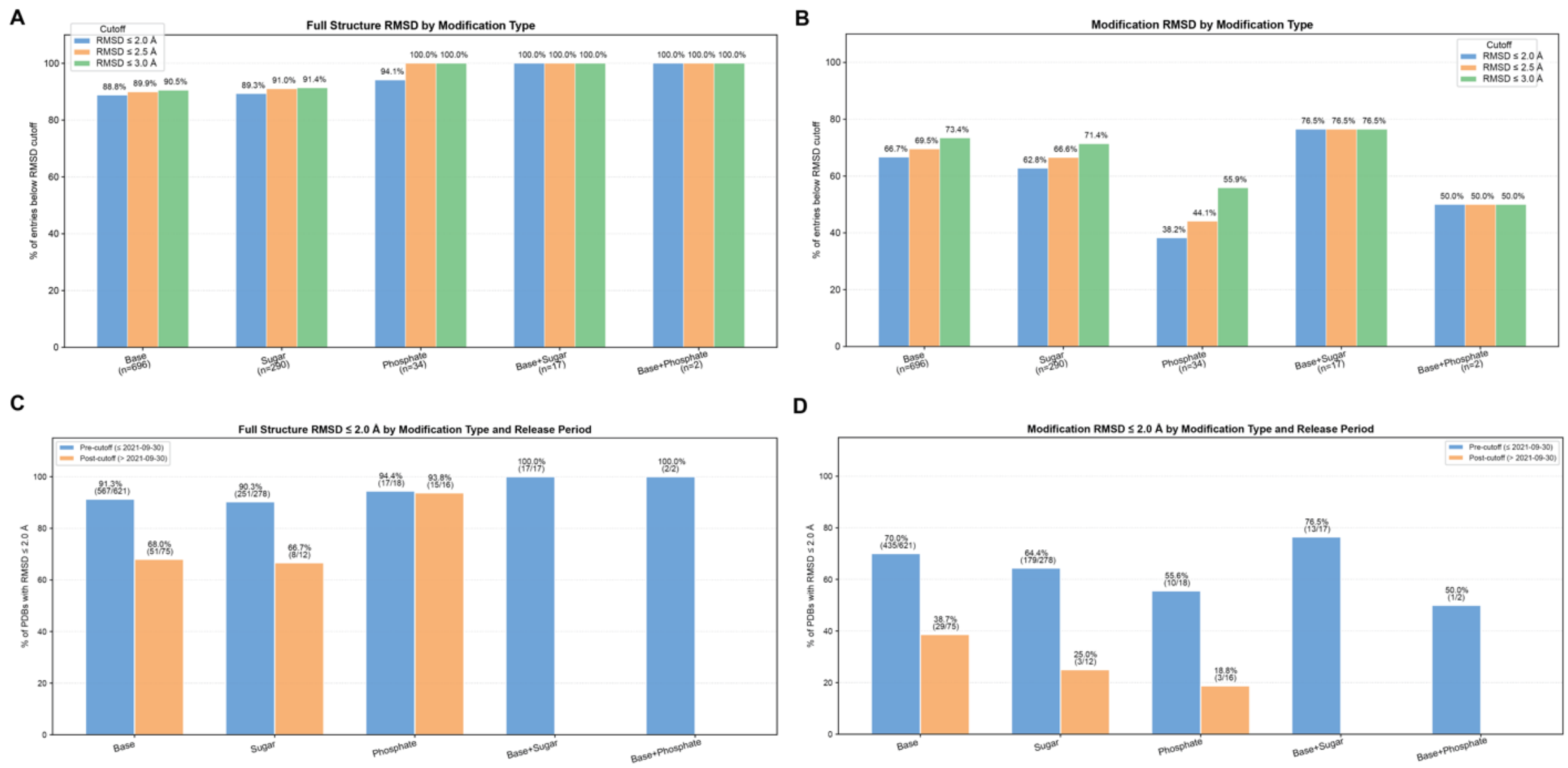

Supplementary Figure 17. Performance of AlphaFold 3 on MODNAP entries pre- and post-training cutoff date by modification type. (A) Percentage of PDB entries with full structure RMSD of AlphaFold 3 models versus experimental structures for all MODNAP entries separately under RMSD threshold values of 2 Å, 2.5 Å, or 3 Å. (B) Percentage of PDB entries with modified nucleotide RMSD of AlphaFold 3 models versus experimental structures for all MODNAP entries separately under RMSD threshold values of 2 Å, 2.5 Å, or 3 Å. (C) Percentage of PDB entries with full structure RMSD of AlphaFold 3 models under RMSD threshold values of 2 Å for MODNAP entries released before (blue, pre-cutoff) or after (orange, post-cutoff) AlphaFold 3's training cutoff date. (D) Percentage of PDB entries with modified nucleotide RMSD of AlphaFold 3 models under RMSD threshold values of 2 Å for MODNAP entries released before (blue, pre-cutoff) or after (orange, post-cutoff) AlphaFold 3's training cutoff date.
